# Colorectal Cancer Cells–Derived Exosomal *PIK3CA* Mutation DNA Promotes Tumor Metastasis by Activating Fibroblast and Affecting Tumor Metastatic Microenvironment

**DOI:** 10.1002/advs.202501792

**Published:** 2025-05-08

**Authors:** Rui Wang, Wanming Li, Yuqiong Lv, Wei Ba, Ying Jiang, Xiaoshuai Li, Jin Fang

**Affiliations:** ^1^ Department of Cell Biology Key Laboratory of Cell Biology Ministry of Public Health Key Laboratory of Medical Cell Biology Ministry of Education China Medical University Shenyang 110122 China; ^2^ Department of Stem Cells and Regenerative Medicine Key Laboratory of Cell Biology Ministry of Public Health Key Laboratory of Medical Cell Biology Ministry of Education China Medical University Shenyang 110122 China; ^3^ Department of Blood Transfusion Shengjing Hospital of China Medical University Shenyang 110000 China

**Keywords:** cancer‐associated fibroblasts, colorectal cancer, exosomal PIK3CA^H1047R^ mutation DNA, peritumoral microenvironment, tumor metastatic microenvironment

## Abstract

Exosomes participate in the formation of the tumor metastatic microenvironment (TME) by delivering tumor‐specific substances. However, current studies mostly focus on exosomal RNA and proteins and lack an in‐depth exploration of exosomal DNA. It is discovered that *PIK3CA* ^H1047R^ mutant DNA in colorectal cancer (CRC) cell‐derived exosomes can be delivered into recipient fibroblasts, where they are transcribed and translated, ultimately leading to the activation of fibroblasts into cancer‐associated fibroblasts (CAFs) through interaction with the endogenous P85 regulatory subunit of the phosphatidylinositol 3‐kinase (PI3K) pathway. CAFs have facilitated tumor cell migration in vitro and promote lung metastasis in vivo by secreting elevated levels of IL6. Additionally, the *PIK3CA* ^H1047R^ mutation is detected in CAFs at both the primary and metastatic sites, suggesting that it may play a role in promoting metastasis by influencing the TME. Moreover, patients with CRC harboring the *PIK3CA* ^H1047R^ mutation and exhibiting elevated levels of IL6 are significantly more likely to metastasize. These findings suggest that the simultaneous detection of serum‐derived exosomal *PIK3CA* ^H1047R^ mutation and serum IL6 secretion may serve as a promising diagnostic and prognostic tool for CRC and simultaneous targeting of *PIK3CA* ^H1047R^ mutation and IL6 may serve as a novel approach for the treatment of CRC.

## Graphical abstract

*PIK3CA* ^H1047R^ DNA in colorectal cancer (CRC) cell‐derived exosomes can be delivered into recipient fibroblasts, where it is transcribed and translated, leading to the activation of fibroblasts into cancer‐associated fibroblasts, which secrete elevated levels of IL6. The simultaneous detection and targeting of *PIK3CA* ^H1047R^ mutation and IL6 may serve as a promising diagnostic and treatment tool for CRC.

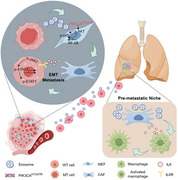

## 1 Introduction

Colorectal cancer (CRC) is a major cause of global morbidity and mortality, highlighting the urgent need for improved diagnostic and treatment strategies.^[^ ^1^ ^]^ Metastasis is a pivotal phase in the advancement of tumors, the substantial challenge in tumor treatment, and the leading cause of death in patients with CRC.^[^ ^2^ ^]^ The tumor microenvironment (TME) plays a critical role in tumor cell proliferation, epithelial‐mesenchymal transition (EMT), and peritumoral microenvironment (PMN), ultimately leading to tumor metastasis.^[^ ^3^ ^]^

The TME comprises various components, such as cancer‐associated fibroblasts (CAFs), inflammatory immune cells, mesenchymal stem cells, endothelial cells of the blood and lymphatic systems, and the extracellular matrix.^[^ ^4^ ^]^ CAFs are one of the main cell types in TME. Extensive research have demonstrated the recruitment of fibroblasts during tumor evolution and their transformation into CAFs, which actively contribute to the restructuring of the TME to promote tumor metastasis. Ferri‐Borgogno^[^ ^5^ ^]^ et al. discovered that ovarian tumor cells can restructure the TME by inducing the transformation of fibroblasts into CAFs. Hsu^[^ ^6^ ^]^ et al. demonstrated that KRAS‐mutated CRC cells could enhance angiogenesis by stimulating fibroblasts to transform into CAFs. Jenkins^[^ ^7^ ^]^ et al. observed that breast tumor cells trigger an immunosuppressive reaction through fibroblast activation. However, the complexity of the interactions between tumor cells and TME hinders a comprehensive understanding of the modes and mechanisms of interaction underlying tumor metastasis.

Exosomes are small double‐membrane vesicles ranging from 30 to 150 nm, are released by cells, and carry biologically active components such as DNA, RNA, proteins, and lipids.^[^ ^8^, ^9^ ^]^ Tumor‐derived exosomes have been suggested to transport tumor‐specific substances to fibroblasts during tumor metastasis, thereby influencing homeostasis. Zhang^[^ ^10^ ^]^ et al. demonstrated that bladder tumor‐derived exosomes activate fibroblasts to CAFs by delivering miR‐146a‐5p to promote tumor metastasis. Li^[^ ^11^ ^]^ et al. found that breast tumor‐derived exosomes promote fibroblast activation by delivering Surviving to affect tumor metastasis. However, existing research has predominantly focused on the role of exosomal RNA or proteins, with limited attention on abnormal exosomal DNA. In 2014, Thakur^[^ ^12^ ^]^ et al. discovered that exosomes contain a considerable amount of double‐stranded DNA (dsDNA), especially in those from tumorous origins. Yang^[^ ^13^ ^]^ et al. have identified *KRAS* and *TP53* mutations in exosomes derived from pancreatic ductal adenocarcinoma cells. Bernard^[^ ^14^ ^]^ et al. observed that exosomal DNA shares the mutational status of its parental cells and an elevated occurrence of mutant *KRAS* within exosomes is linked to unfavorable prognosis and survival rates. Detecting *KRAS* and *EGFR* mutations in serum exosomes may have greater clinical significance than detecting circulating tumor DNA.^[^ ^15^, ^16^ ^]^ However, research on exosomal DNA has primarily focused on surveillance of tumorigenesis and development, and the precise mechanism of action of exosomal DNA in tumor progression remains to be fully elucidated.

Moreover, exosomal DNA can enter the recipient cells. Fischer^[^ ^17^ ^]^ et al. demonstrated that human bone marrow‐derived mesenchymal stromal cell‐derived exosomal *Arabidopsis thaliana*–DNA can perform RNA transcription and protein translation in recipient cells. Lee^[^ ^18^ ^]^ et al. found that intestinal epithelial cell‐derived exosomal *HRAS* is stabilized in RAT‐1 cells. Zhao^[^ ^19^ ^]^ et al. also discovered that the exosome‐packaged dsDNA modulates immune functions. Furthermore, in tumor cells, Balaj^[^ ^20^ ^]^ et al. have documented that exosomes harbor 5ʹ promoter regions, 3ʹ untranslated regions, and active reverse transcriptional transposons. They also observed that glioma cell‐derived exosomal *c‐Myc* could be transferred to recipient cells and integrated into the genome. Chang^[^ ^21^ ^]^ et al. found that *KRAS*‐dependent tumor cells promote cell survival by secreting exosomes. However, it is unclear whether this functional change is caused by exosomal DNA delivery.

Class IA phosphatidylinositol 3‐kinase (PI3K) are heterodimeric lipid kinases comprising a p110 catalytic subunit and a p85 regulatory subunit.^[^ ^22^ ^]^ The PIK3CA, responsible for encoding the p110α catalytic subunit of PI3K, exhibits a mutation frequency of over 25% in patients with CRC, and the majority of *PIK3CA* mutations are concentrated at three specific sites: E542K and E545K in the helix domain, and H1047R in the kinase domain.^[^ ^23^ ^]^ The *PIK3CA* ^H1047R^ mutation is also associated with CRC metastasis.^[^ ^24^, ^25^ ^]^ However, the role of *PIK3CA* ^H1047R^ mutation in CRC metastasis remains unclear. Additionally, metastatic tumors have a higher exosomal *PIK3CA* ^H1047R^ mutation frequency,^[^ ^26^ ^]^ and *PIK3CA* ^H1047R^ mutated CRC cells can activate the transformation of fibroblasts into CAFs.^[^ ^27^ ^]^ However, it is unclear whether this is mediated by exosomes.

In this study, we applied the CRISPR/Cas9 technology to CRC cells harboring the *PIK3CA* ^H1047R^ mutation to establish a *PIK3CA* wild type (WT) cell line. Exosomes were isolated from the two cell types and used to treat fibroblasts. The CRC cell‐derived exosomal *PIK3CA* ^H1047R^ mutation was transcribed, translated in the recipient fibroblasts, and persisted in the passaged cells. Exosomal *PIK3CA* ^H1047R^ mutation could transform fibroblasts into CAFs, facilitating metastasis of tumor cells. Our findings elucidate the important role of the horizontal transmission of *PIK3CA* ^H1047R^ mutation via exosomes derived from CRC cells in influencing TME homeostasis, as well as the potential molecular mechanism underlying the promotion of CRC metastasis.

## 2 Result

### 2.1 LS174T Cells with the *PIK3CA* ^H1047R^ Mutation were Gene‐Edited to *PIK3CA* ^WT^ and Functionally Characterized

To examine the role of exosomal *PIK3CA* ^H1047R^ mutation in the development of CRC, CRISPR/Cas9 technology was employed to introduce a single base change (G→A) at nucleotide 3140 in LS174T cells, converting the codon for Arg‐1047 to His. As a result, the LS174T‐*PIK3CA* ^WT^ cell line was established, where the original LS174T‐*PIK3CA* ^H1047R^ cells were referred to as MT cells, and gene‐edited LS174T‐*PIK3CA* ^WT^ cells were referred to as WT cells. Thus, the genetic backgrounds of the two cell types were identical except for the mutation site. Sanger sequencing (Figure S1A, Supporting Information) and western blotting (Figure S1B, Supporting Information) were used to identify the mutation sites following gene editing. Importantly, MT cells showed stronger proliferative (Figure S1C, Supporting Information) and migratory abilities (Figure S1D,E, Supporting Information) in vitro compared with those of WT cells. *PIK3CA* ^H1047R^ mutation causes constitutive activation of PI3K. To compare the PI3K activity in WT and MT cells, we measured the intracellular levels of phosphorylated AKT (pAKT), one of the most important downstream substrates of the PI3K signaling pathway. Consistent with the expected results, MT cells showed higher levels of pAKT than WT cells (Figure S1F, Supporting Information).

Furthermore, a xenograft model was established by subcutaneously injecting 4 × 10^6 ^WT and MT cells into the left and right sides of BALB/c mice to examine the proliferative capacity of these cells in vivo, as shown in Figure S1G(a) (Supporting Information). The results demonstrated that MT cells exhibited significant tumorigenicity compared with that in WT cells (Figure S1G(b), Supporting Information), as evidenced by the larger size (Figure S1H, Supporting Information) and weight (Figure S1I, Supporting Information) of the tumors formed. To investigate the metastatic potential of WT and MT cells in vivo, 4 × 10^6^ WT and MT cells were intravenously injected into BALB/c mice. After 60 days, the mice were euthanized and each organ was extracted and subjected to gross observations. The injected MT cell group exhibited the formation of metastatic foci in the lungs in three of the four mice (3/4), whereas the injected WT cell group did not (Figure S1J, Supporting Information). Analysis of the metastatic capability using hematoxylin and eosin (HE) staining indicated a higher number of metastatic foci in the lungs of the group injected with MT cells (Figure S1K(a,b), Supporting Information). These results suggest that the gene‐edited WT cells have altered functions both in vitro and in vivo, indicating that we successfully constructed a WT cell line.

### 2.2 WT and MT Cell‐Derived Exosomes Contain the Same *PIK3CA* Mutation Status as Parental Cells

**Figure 1:**
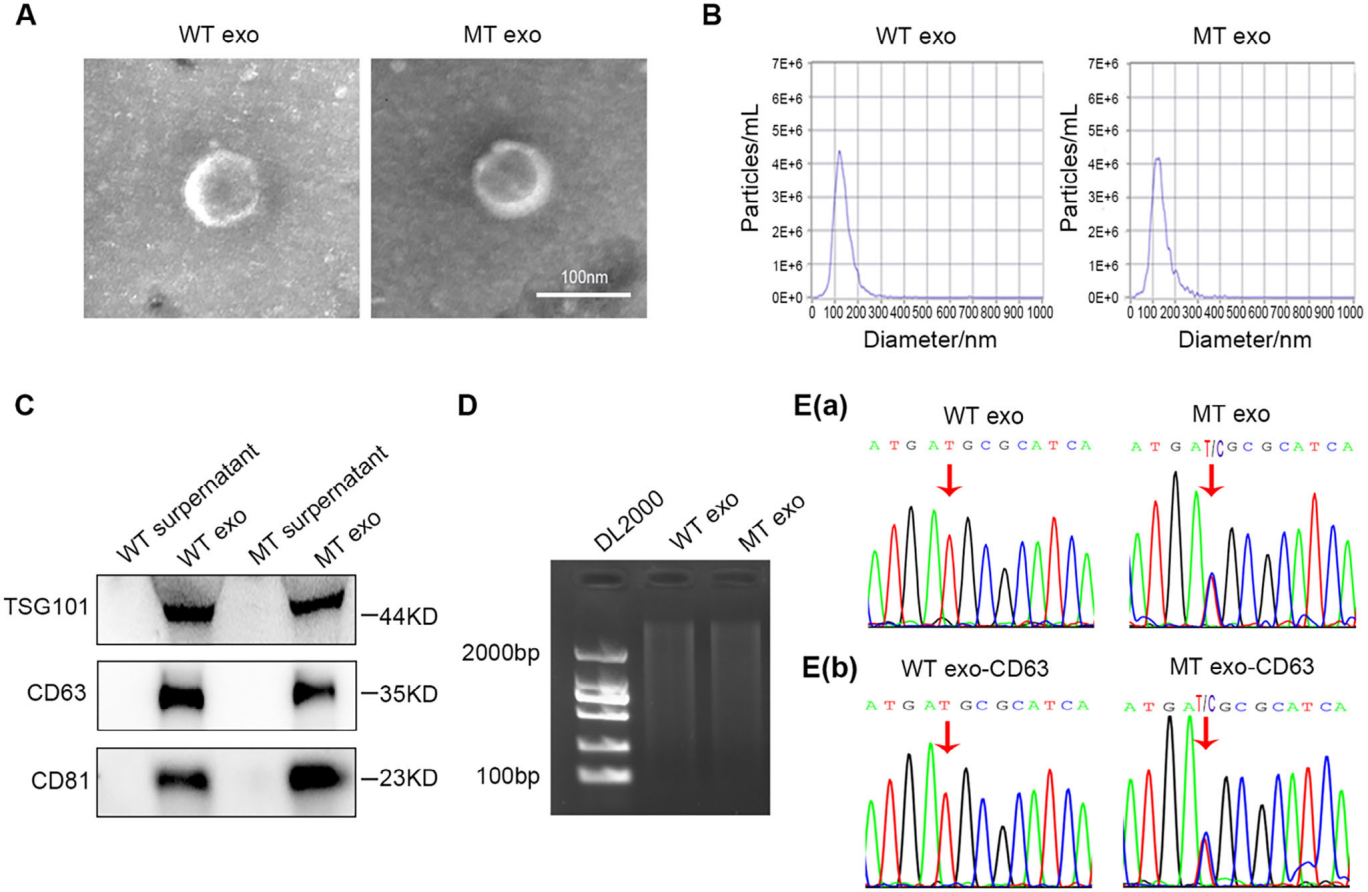
WT and MT cell‐derived exosomes contain the same *PIK3CA* mutation state as parental cells. A) Electron microscopy image of exosomes exacted from WT and MT cells. Scale bar, 100 nm. B) Exosome size distribution from WT and MT cells measured by NTA. C) Western blot analysis of the expression of TSG101, CD63, CD81 of exosomes derived from WT and MT cells. D) DNA in WT and MT cell‐derived exosomes on 1% agarose gel. DNA ladder, DL2000. E) a) Sanger sequencing analysis of *PIK3CA* ^H1047R^ mutation in WT and MT cell‐derived exosomes. E) b) Sanger sequencing analysis of *PIK3CA* ^H1047R^ mutation in CD63‐positive WT and MT cell‐derived exosomes.

To investigate whether the WT and MT cell‐derived exosomes contain the same *PIK3CA* mutation as the parental cells, exosomes were isolated from the supernatants of WT and MT cells using ultracentrifugation. The isolated exosomes were then observed under a transmission electron microscope, revealing the presence of round vesicles ≈100 nm in size and cup‐shaped structures surrounded by a bilayer membrane (**Figure** **1**A). The size of the exosomes was confirmed using nanoparticle tracking analysis (NTA) (Figure 1B). Moreover, the expression of the characteristic exosome markers TSG101, CD63, and CD81 further confirmed that the isolated components were exosomes (Figure 1C). To avoid interference from the DNA attached to the surface of the exosomes, we pretreated the exosomes with DNase I prior to application. Subsequently, agarose gel electrophoresis demonstrated the successful extraction of DNA from exosomes derived from both WT and MT cells, which were mainly distributed in fragments with an approximate range of 100–2000 bp (Figure 1D).

To clarify the controversy regarding the presence of dsDNA in exosomes, we used agarose gel electrophoresis to identify DNA in exosomes originating from different CRC cells. Our findings revealed that the abundance of DNA in the exosomes varied. Specifically, we observed that HCT116, SW480, LS174T, and HT29 cell‐derived exosomes exhibited a higher abundance of DNA than that in RKO and LOVO cell‐derived exosomes (Figure S2A, Supporting Information). We also used agarose gel electrophoresis to examine *PIK3CA* DNA in exosomes derived from HCT116, RKO, and LS174T cell lines harboring the *PIK3CA* ^H1047R^ mutation, demonstrating that exosomes derived from harboring the *PIK3CA* ^H1047R^ mutation cells exhibited *PIK3CA* (Figure S2B, Supporting Information). Droplet digital PCR (ddPCR) analysis demonstrated that exosomes derived from LS174T cells exhibited a higher mutation frequency of *PIK3CA* ^H1047R^ than that in exosomes derived from the other two cell lines (**Table** **1**). Therefore, clarification is needed to determine whether the specific gene template quantities in exosomes from cells with similar DNA abundances are comparable. Figure S2A,B (Supporting Information) and Table 1 illustrate that, while the total DNA content of HCT116 cells was comparable to that of LS174T cells, there was a disparity in the mutation frequency of *PIK3CA* ^H1047R^. This indicates that exosomal DNA packaging may be selective. These results further demonstrate that exosomes originating from MT cells exhibit elevated DNA content, particularly in relation to the mutation frequency of *PIK3CA* ^H1047R^, facilitating subsequent research. Sanger sequencing demonstrated that exosomes derived from MT cells harbored the *PIK3CA* ^H1047R^ mutation, whereas exosomes derived from WT cells did not (Figure 1E(a)). This suggested that the exosomes retained the same genetic information as their parent cells.

**Table 1:** Mutational analysis of *PIK3CA* gene in cell‐derived exosome‐DNA by ddPCR.

Cell type	Gene	Position	Mutation frequency [%]
HCT116	*PIK3CA*	H1047R	46.29
LS174T	*PIK3CA*	H1047R	51.52
RKO	*PIK3CA*	H1047R	36.44

To further validate that the extracted DNA was derived from exosomes rather than from other extracellular vesicles, we employed a co‐incubation approach using magnetic beads coated with an antibody specific to the exosome marker CD63. Subsequent DNA extraction from the co‐incubated exosomes confirmed that the extracted DNA originated from CD63‐positive exosomes. Sanger sequencing analysis demonstrated that the *PIK3CA* ^H1047R^ mutation was present in CD63‐positive exosomes derived from MT cells (Figure 1E(b)), providing further evidence that the *PIK3CA* ^H1047R^ mutation in MT cell‐derived exosomes was identical to that in the parental cells.

### 2.3 Horizontal Transmission of *PIK3CA* ^H1047R^ Mutation in MT Cell‐Derived Exosomes to Fibroblasts

**Figure 2:**
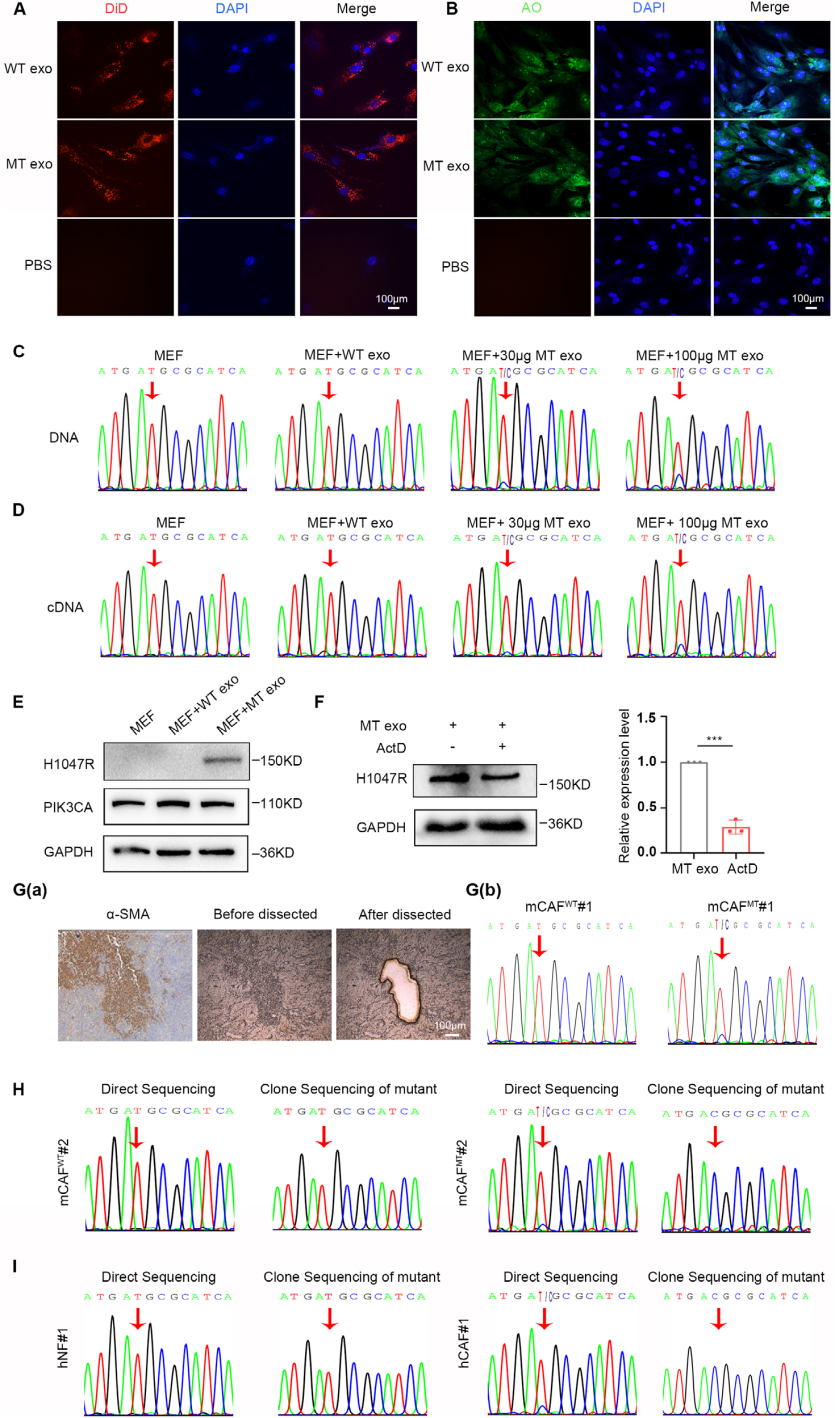
Horizontal transmission of *PIK3CA* ^H1047R^ mutation in MT cell‐derived exosomes to fibroblasts. A) Image of DiD‐stained exosome from WT and MT cells transfer to MEFs. Scale bar, 100 µm. B) Image of AO‐stained exosomal DNA from WT and MT cells transfer to the nucleus of MEFs. Scale bar, 50 µm. C) Sanger sequencing analysis of *PIK3CA* ^H1047R^ mutation in DNA of MEFs co‐incubated with WT and MT cell‐derived exosomes for 24 h. D) Sanger sequencing analysis of *PIK3CA* ^H1047R^ mutation in cDNA of MEFs co‐incubated with WT and MT cell‐derived exosomes for 72 h. E) Western blotting analysis of H1047R protein in MEFs co‐incubated with WT and MT cell‐derived exosomes for 72 h. F) Western blotting analysis of H1047R expression after ActD (80 nm) was treated. Quantitative analysis is shown in the right panel. Error bars, mean ± SD. Two‐sided Student's *t*‐test. *** *P* < 0.0001, *n *= 3. G) a) Representative images that microdissected on a Leica LMD6000 laser fiber cutting instrument of paraffin section from tumors of mice with WT and MT cells subcutaneously injection, the consecutive sections with α‐SMA‐positive immunohistochemistry served as a control. Scale bar, 100 µm. G) b) Sanger sequencing analysis of *PIK3CA* ^H1047R^ mutation in the microdissected CAFs. H) Sanger sequencing of the direct sequencing and T‐A clonal sequencing analysis of *PIK3CA* ^H1047R^ mutation in fifth‐passage mCAFs. I) Sanger sequencing of the direct sequencing and T‐A clonal analysis of *PIK3CA* ^H1047R^ mutation in hCAFs and hNFs from one set of paired patients with CRC.

Mouse embryonic fibroblasts (MEFs) were isolated from mouse embryos and selected as recipient fibroblasts. To assess exosome delivery, we labeled exosomes derived from both WT and MT cells using the cell membrane red fluorescent probe, DiD. Following a 24h co‐incubation, fluorescence microscopy revealed the presence of red punctate signals within MEFs (**Figure** **2**A), indicating the successful delivery of labeled exosomes derived from WT and MT cells to MEFs. To further confirm that exosomal DNA was internalized by MEFs, exosomal DNA was stained with acridine orange (AO). Following a 24 h co‐incubation, green DNA signals were observed within the nuclei of MEFs by super‐resolution confocal microscopy, consistent with the DiD staining results for exosomes (Figure 2B).

To further confirm that the *PIK3CA* ^H1047R^ mutation in MT cell‐derived exosomes was horizontally to delivered to MEFs, MEFs were co‐incubated with exosomes derived from MT cells for 72 h. Sanger sequencing indicated that MEFs treated with MT cell‐derived exosomes contained the *PIK3CA* ^H1047R^ mutation in a dose‐dependent manner, suggesting that the *PIK3CA* ^H1047R^ mutation in MT cell‐derived exosomes can be horizontally delivered to MEFs (Figure 2C). Due to DNA transfer from exosomes to the nuclei of MEFs, our study aimed to investigate the transcription and translation of the *PIK3CA* ^H1047R^ mutation in MEFs. RNA and protein samples were collected from MEFs treated with various exosomes for 72 h. Following the conversion of RNA into cDNA, sanger sequencing revealed the presence of the *PIK3CA* ^H1047R^ mutation within the cDNA of MEFs treated with MT cell‐derived exosomes in a dose‐dependent manner (Figure 2D). Furthermore, western blot analysis confirmed the expression of PIK3CA^H1047R^ protein in MEFs with MT cell‐derived exosomes (Figure 2E), suggesting that the horizontally transferred *PIK3CA* ^H1047R^ mutation can undergo transcription and translation processes in MEFs. Notably, such signals were still present in passaged cells, albeit at diminished levels, indicating that the exosomal *PIK3CA* ^H1047R^ mutation may be stable in MEFs (Figure S3A, B, C, Supporting Information). Whole exome sequencing (WES) further corroborated the presence of the *PIK3CA* ^H1047R^ mutation in the MEFs genome (Table S1, Supporting Information), leading to the production of the H1047R protein through transcription and translation in MEFs.

To investigate whether the *PIK3CA* ^H1047R^ mutation in exosomes was selectively delivered to specific cells or distributed randomly across different cell types, we applied MT cell exosomes at the same concentration to various cell lines, including human macrophage THP‐1, human epidermal HaCaT, MEFs, mouse macrophages Raw264.7, human umbilical vein endothelial HUVECs, and WT cells. None of these cells expressed H1047R in the background and subsequent western blotting revealed that the *PIK3CA* ^H1047R^ mutation in MT cell‐derived exosomes was predominantly delivered to MEFs and HUVECs, with a lesser extent of delivery observed in THP‐1, Raw264.7, and WT cells, and minimal delivery to HaCaT cells (Figure S3D, Supporting Information). Our findings indicate that the transmission of the *PIK3CA* ^H1047R^ mutation via MT cell exosomes is selective rather than random across different cell types, with fibroblasts receiving the highest levels of the mutation.

To exclude the effects of exosomal RNA and protein delivery, MEFs were co‐incubated with MT cell‐derived exosomes and the transcriptional inhibitor actinomycin D (ActD) for 72 h. The results showed that the H1047R expression level was reduced by ≈75% compared with that in the non‐ActD‐treated group (Figure 2F), suggesting that H1047R expression in MEFs was dominantly due to exosomal *PIK3CA* ^H1047R^ DNA delivery. MEFs are of murine origin, therefore, we also used human foreskin fibroblasts (hFbs) as the recipient cell to observe whether the transmission of the exosomal *PIK3CA* ^H1047R^ mutation has a similar process in human cells. Sanger sequencing analysis also revealed that *PIK3CA* ^H1047R^ mutation in exosomes derived from MT cells could be horizontally transferred to hFbs (Figure S4A,B, Supporting Information).

These experiments demonstrate that the *PIK3CA* ^H1047R^ mutation in exosomes derived from MT cells was horizontally transmitted in vitro. We used paraffin sections from WT and MT cell xenograft model tissues for in vivo experiments. CAFs of these tissues were isolated using laser capture microdissection (LCM), while the consecutive sections with α‐SMA‐positive immunohistochemistry served as a control (Figure 2G(a)). The *PIK3CA* ^H1047R^ mutation was detected in the CAFs of MT cell‐derived tumor tissues using sanger sequencing (Figure 2G(b)). We initially isolated mouse CAFs (mCAFs) from the tumors of mice subcutaneously injected with WT and MT cells. The morphology of primary mCAFs was predominantly fibroblastic (Figure S5A, Supporting Information). Sanger sequencing confirmed the *PIK3CA* ^H1047R^ mutation in mCAFs^MT^, and which was further validated through T‐A clonal sequencing (Figure 2H). To determine whether there was a *PIK3CA* ^H1047R^ mutation in the CAFs of patients with CRC, we collected CAFs from ten patients with CRC, comprising five sets of paired CAFs and human normal fibroblasts (hNFs), where the hNFs were located 20 cm away from the tumor tissue. Western blotting was further identified the specific markers in hCAFs and hNFs (Figure S5B, Supporting Information). Sanger sequencing of hCAFs revealed the presence of the *PIK3CA* ^H1047R^ mutation in four out of ten patients with CRC (4/10), whereas these mutations were absent in hNFs. A representative sequencing image of hCAFs and hNFs from one set of paired samples (Figure 2I). Furthermore, immunohistochemical staining of paraffin sections from patients with CRC tissues revealed the expression of H1047R in CAFs (Figure S6, Supporting Information). These findings indicate that horizontal transmission of *PIK3CA* ^H1047R^ mutation in MT cell‐derived exosomes to fibroblasts is plausible and occurs in patients with CRC.

### 2.4 Exosomal *PIK3CA* ^H1047R^ Mutation Activated MEFs into CAFs

**Figure 3:**
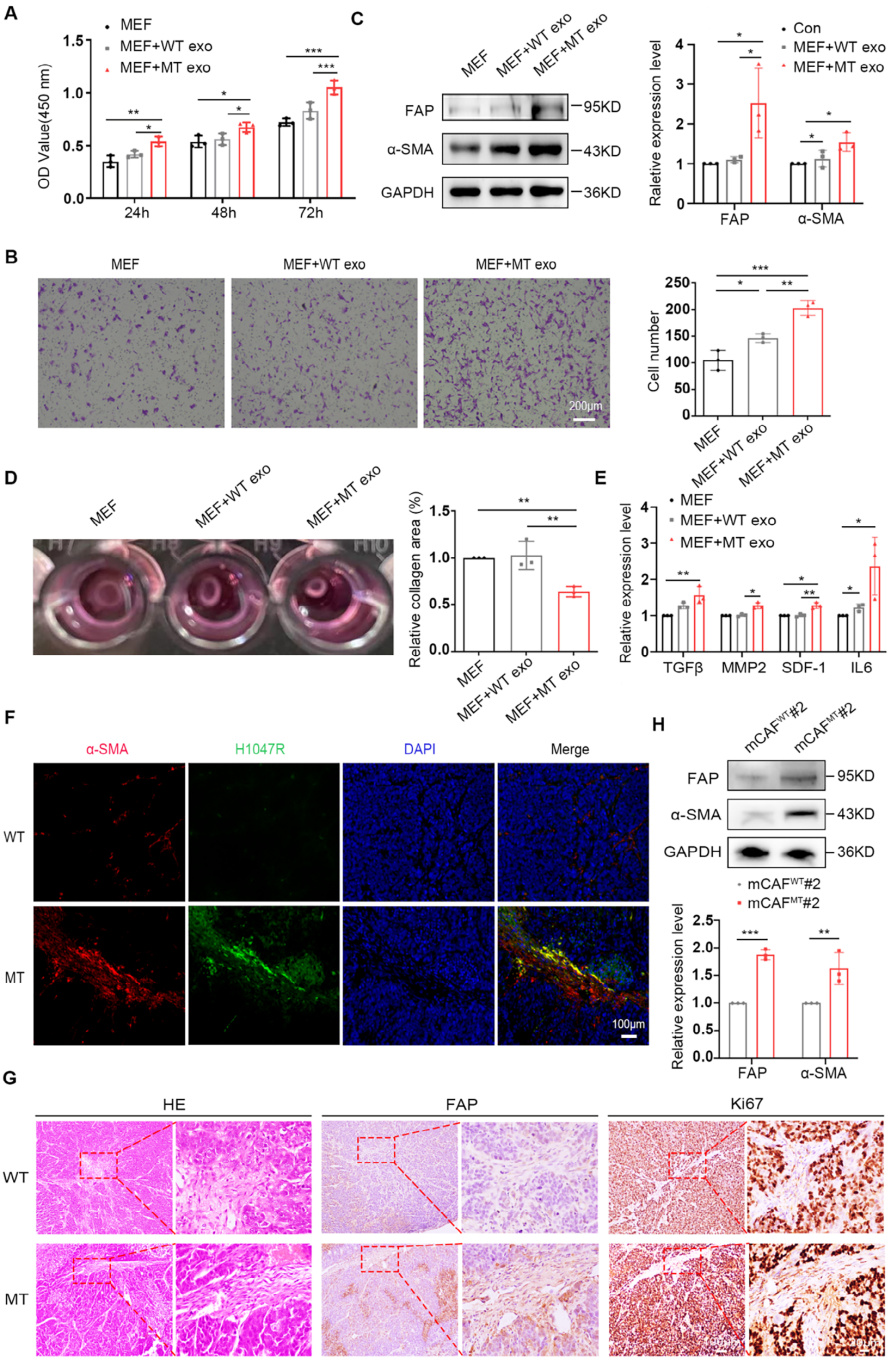
Exosomal *PIK3CA* ^H1047R^ mutation activates MEFs into CAFs. A) CCK8 assay to determine the proliferation viability of MEFs treated with WT or MT cell‐derived exosomes. Error bars, mean ± SD. Two‑way ANOVA with multiple comparisons. *** *P* < 0.0001, *n *= 3. B) Transwell assay to determine the migration viability of MEFs treated with WT or MT cell‐derived exosomes for 72 h. Quantitative analysis is shown in the right panel. Error bars, mean ± SD. One‑way ANOVA with multiple comparisons. * *P* < 0.01, ** *P* < 0.001,*** *P* < 0.0001, *n *= 3. Scale bar, 200 µm. C) Western blot analysis of CAFs markers FAP and α‐SMA in MEFs treated with WT or MT cell‐derived exosomes for 72 h. Quantitative analysis is shown in the right panel. Error bars, mean ± SD. Two‑way ANOVA with multiple comparisons. * *P* < 0.01, *n *= 3. D) Collagen contraction assay of the blank control and WT or MT cell‐derived exosomes treated MEFs. The area change of the gels was recorded at 72 h. Representative images are shown in the right panel. Error bars, mean ± SD. One‑way ANOVA with multiple comparisons. ** *P* < 0.001, *n *= 3. E) Real‐time PCR analysis of TGFβ, MMP2, SDF‐1, and IL6 expression in MEFs treated with WT or MT cell‐derived exosomes for 72 h. Error bars, mean±SD. Two‑way ANOVA with multiple comparisons. * *P* < 0.01, ** *P* < 0.001, *n *= 3. F) Immunofluorescence analysis of H1047R and α‐SMA expression insections of tumors from mice with WT and MT cells subcutaneously injection. Scale bar, 100 µm. G) HE staining and immumohistochemical analysis of FAP and Ki67 expression in serial paraffin sections of tumors from mice with WT and MT cells subcutaneously injection. Scale bar, 100 µm (left) and 20 µm (right). H) Western blot analysis of FAP and α‐SMA expression in mCAFs. Quantitative analysis is shown in the below panel. Error bars, mean ± SD. Two‑way ANOVA with multiple comparisons. ** *P* < 0.001, *** *P* < 0.0001, *n *= 3.

CAFs are fibroblasts in an activated state with the ability to significantly promote tumorigenesis.^[^ ^28^ ^]^ To elucidate whether the horizontal transmission of the *PIK3CA* ^H1047R^ mutation in MT cell‐derived exosomes activates the transformation of MEFs into CAFs, MEFs were treated with the two types of exosomes for 72 h. Compared with exosomes derived from WT cells, MEFs co‐incubated with exosomes derived from MT cells exhibited a significant increase in the proliferative capacity (**Figure** **3**A), migratory capacity (Figure 3B), and expression levels of CAFs markers FAP and α‐SMA (Figure 3C). These suggest that the horizontal transfer of *PIK3CA* ^H1047R^ mutation through MT cell‐derived exosomes has the potential to induce MEF to CAF transformation. Additionally, CAFs are characterized by heightened contractility and the ability to secrete cytokines.^[^ ^28^ ^]^ To further corroborate our findings, collagen contraction provided evidence that the contractile ability of MEFs was enhanced after co‐incubation with MT cell‐derived exosomes (Figure 3D). Furthermore, the results indicated elevated levels of cytokines secretion in MEFs including transforming growth factor‐β (TGFβ), matrix metallopeptidase 2 (MMP2), stromal cell‐derived factor 1(SDF‐1), IL6 after co‐incubation with MT cell‐derived exosomes (Figure 3E). This validation was further confirmed using WT and MT‐overexpressing MEFs, which yielded consistent results (Figure S7A–D, Supporting Information). To validate the outcomes of the in vitro experiments, we examined paraffin sections from the tumors of mice subcutaneously injected with WT and MT cells. Immunofluorescence analysis revealed co‐localization of H1047R and α‐SMA at the same site (Figure 3F). These findings suggest fibroblast activation at the horizontal transmission of the *PIK3CA* ^H1047R^ site, supporting the notion that fibroblast activation is driven by the horizontal transmission of *PIK3CA* ^H1047R^ mutation. Immumohistochemical of FAP and Ki67 in the same field revealed high expression levels of FAP and Ki67 in the MT group (Figure 3G). Additionally, the *PIK3CA* ^H1047R^ mutation promoted fibroblast activation in vivo. We initially isolated mCAFs and conducted western blot assays, which confirmed that mCAFs^MT^ exhibited more pronounced activation characteristics than mCAFs^WT^ (Figure 3H). Collectively, these findings indicated that exosomes derived from MT cells facilitated the activation of fibroblasts through horizontal transmission of the *PIK3CA* ^H1047R^ mutation both in vitro and in vivo.

### 2.5 Exosomal *PIK3CA* ^H1047R^ Mutation is Associated with CRC Metastasis

**Figure 4:**
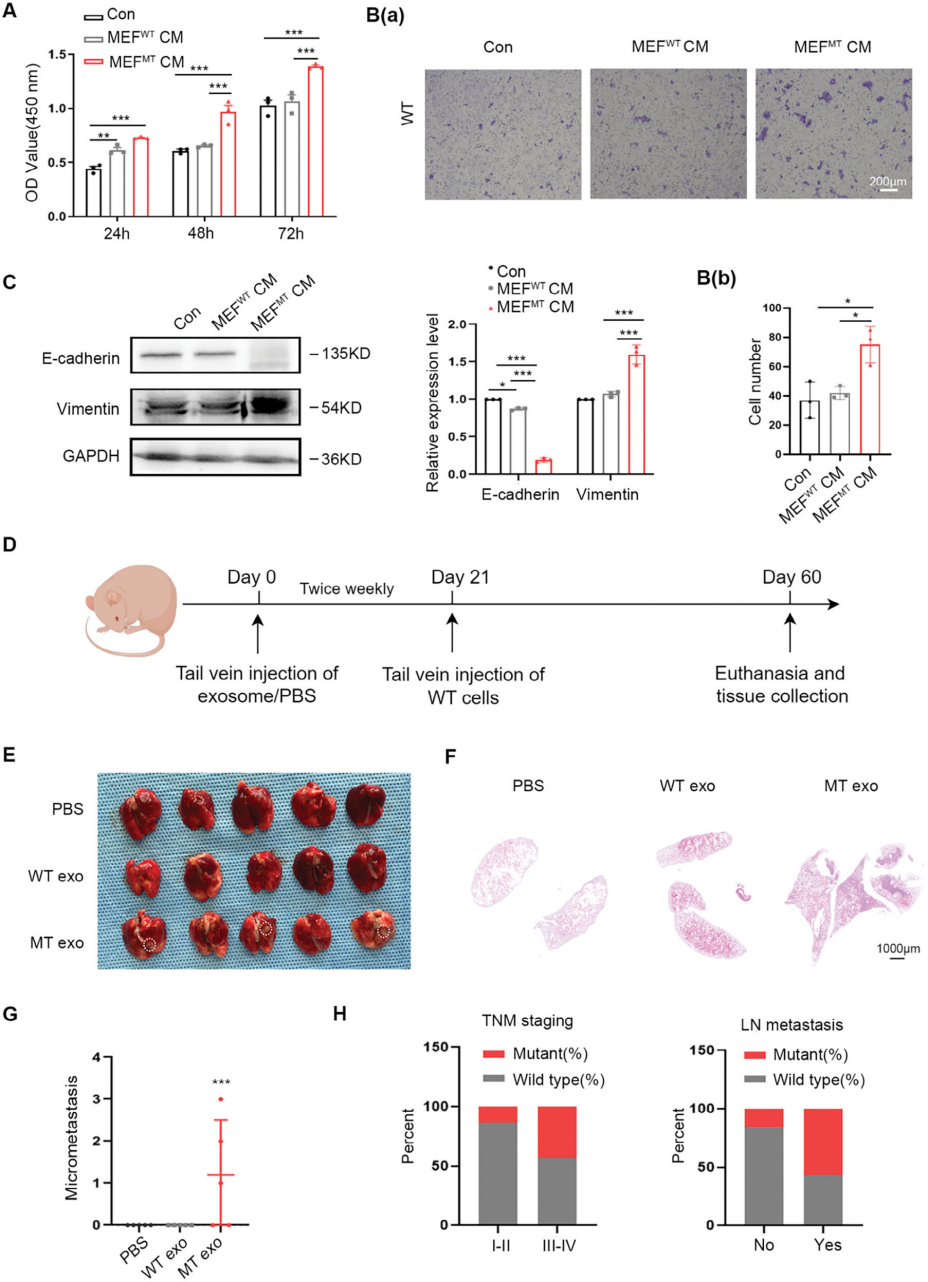
Exosomal *PIK3CA* ^H1047R^ mutation is associated with CRC metastasis. A) CCK8 assay to determine the proliferation viability of WT cells treated with the different indicated MEFs CM. Error bars, mean ± SD. Two‑way ANOVA with multiple comparisons. ** *P* < 0.001, *** *P *< 0.0001, *n *= 3. B) a) Transwell assay to determine the migration viability of WT cells treated with different indicated MEFs CM for 72 h. Scale bar, 200µm. B) b) Quantitative analysis of B(a). Error bars, mean ± SD. One‑way ANOVA with multiple comparisons. * *P* < 0.01, *n *= 3. C) Western blot analysis of EMT markers E‐cadherin and Vimentin expression in WT cells treated with different MEFs CM for 72 h. Quantitative analysis is shown in the right panel. Error bars, mean ± SD. Two‑way ANOVA with multiple comparisons. * *P* < 0.01, *** *P* < 0.0001, *n *= 3. D) Schematic of the animal experimental design. E) Lung tissues with metastatic nodules from the mice of WT and MT cell‐derived exosomes tail vein injection. White circles were the metastatic foci in the lungs, *n *= 5. F) HE staining of lung tissues. Scale bar, 1000 µm. G) The number of lung metastases lesions was counted using HE staining. Error bars, mean ± SD. One‑way ANOVA with multiple comparisons. *** *P* < 0.0001, *n *= 5. H) Statistical analysis of the percentage of *PIK3CA* ^H1047R^ mutation in TNM staging and LN metastasis in patients with CRC.

CAFs play a pivotal role in paracrine tumor progression. Investigating the impact of exosomal *PIK3CA* ^H1047R^ mutation on CRC metastasis necessitated the collection of conditioned medium (CM) from MEFs treated with exosomes derived from both WT and MT cells. Subsequently, CM was administered to WT cells for 72 h for further analysis. The results depicted in **Figure** **4**A,B(a,b) demonstrated that MEFs^MT^ CM significantly enhanced the proliferation and migration ability of WT cells compared with that in MEFs^WT^ CM. The validity of these findings was confirmed using CM from WT and MT‐overexpressing MEFs, which yielded consistent results (Figure S8A,B(a,b), Supporting Information). EMT facilitates the transition of quiescent epithelial cells into a more mobile and aggressive state and plays a crucial role in CRC development.^[^ ^29^ ^]^ In WT cells, MEFs^MT^ CM downregulated the epithelial marker E‐cadherin and upregulated the mesenchymal marker, Vimentin compared with those in MEFs^WT^ CM (Figure 4C). Validation of the above experiments using CM from WT and MT‐overexpressing MEFs yielded similar results (Figure S8C, Supporting Information).

In addition to the recruitment of tumor cells and the occurrence of EMT at the primary site, exosomes also have the potential to establish a PMN.^[^ ^30^ ^]^ A PMN creates a more permissive microenvironment for metastasis at distant sites, thus facilitating the dissemination of tumor cells from the primary site.^[^ ^31^ ^]^ To investigate the potential role of the exosomal *PIK3CA* ^H1047R^ mutation in PMN formation, BALB/c mice were divided into three groups, of five mice each. The tail vein was used to administer the exosomes/PBS twice per week. A total of 4 × 10^6^ WT cells were then intravenously injected into BALB/c mice pretreated with exosomes/PBS. Continue to inject exosomes/PBS into the tail vein twice per week. A schematic of this process is shown in Figure 4D. After 60 days, the mice were euthanized and each organ was extracted and subjected to thorough observations. The injected MT cell‐derived exosome group exhibited the formation of metastatic foci only in the lungs of three of the five mice (3/5), whereas the injected WT cell group did not (Figure 4E). Assessment of the metastatic foci in the three groups of mice was conducted using HE staining (Figure 4F), which indicated a higher number of metastatic foci in the lungs of the group injected with MT cells (Figure 4G). The exosomal *PIK3CA* ^H1047R^ mutation induced CRC metastasis via the activation of fibroblasts both locally and in distant lung tissue PMN, ultimately facilitating the dissemination of tumor cells from the primary site.

To further evaluate the correlation between the exosomal *PIK3CA* ^H1047R^ mutation and the metastatic capacity in patients with CRC, we evaluated the *PIK3CA* ^H1047R^ mutation state in serum‐derived exosomes from fifty‐five patients with CRC. Following the isolation of serum‐derived exosomes, DNase I treatment was applied, and DNA was extracted for T‐A cloning sequencing, resulting in the acquisition of fifty clones from each sample. Sixteen patients exhibited the *PIK3CA* ^H1047R^ mutation, as depicted in Figure S9 (Supporting Information), with a mutation percentage of 29.1%. Notably, twelve of these sixteen cases (12/16) were observed in TNM stages III–IV, and ten of sixteen cases (10/16) had lymph node (LN) metastasis, which demonstrates statistical significance (**Table** **2**). The statistical details are shown in Figure 4h. Consequently, it can be deduced that the exosomal *PIK3CA* ^H1047R^ mutation is associated with the metastatic capacity of patients with CRC.

**Table 2:** Exosomal *PIK3CA* ^H1047R^ mutation is associated with CRC metastasis.

Characteristic	Total [%]	Wild type	Mutant [%]	*P*‐value
Number of patients	55	39	16 (29.1)	
Age(years) >60 ≤60	28 (51) 27 (49)	20 19	8 (28.6) 8 (29.6)	0.9312
Gender Male Female	33 (60) 22 (40)	21 18	12 (36.4) 4 (18.2)	0.1458
Tumor location Colon Rectum	23 (41.8) 32 (58.2)	18 20	5 (21.7) 12 (37.5)	0.2122
Histological grade Low Middle‐high	17 (30.9) 38 (69.1)	11 28	6 (35.5) 10 (26.3)	0.4981
TNM staging I‐II III‐IV	29 (52.7) 26 (47.3)	25 14	4 (17.2) 12 (46.2)	0.0083**
LN metastasis Yes No	18 (32.7) 37 (67.3)	8 31	10 (55.6) 6 (16.2)	0.0026**

### 2.6 The Horizontal Transmission of Exosomal *PIK3CA* ^H1047R^ Mutation Induces CAFs to Secrete Elevated Levels of IL6

**Figure 5:**
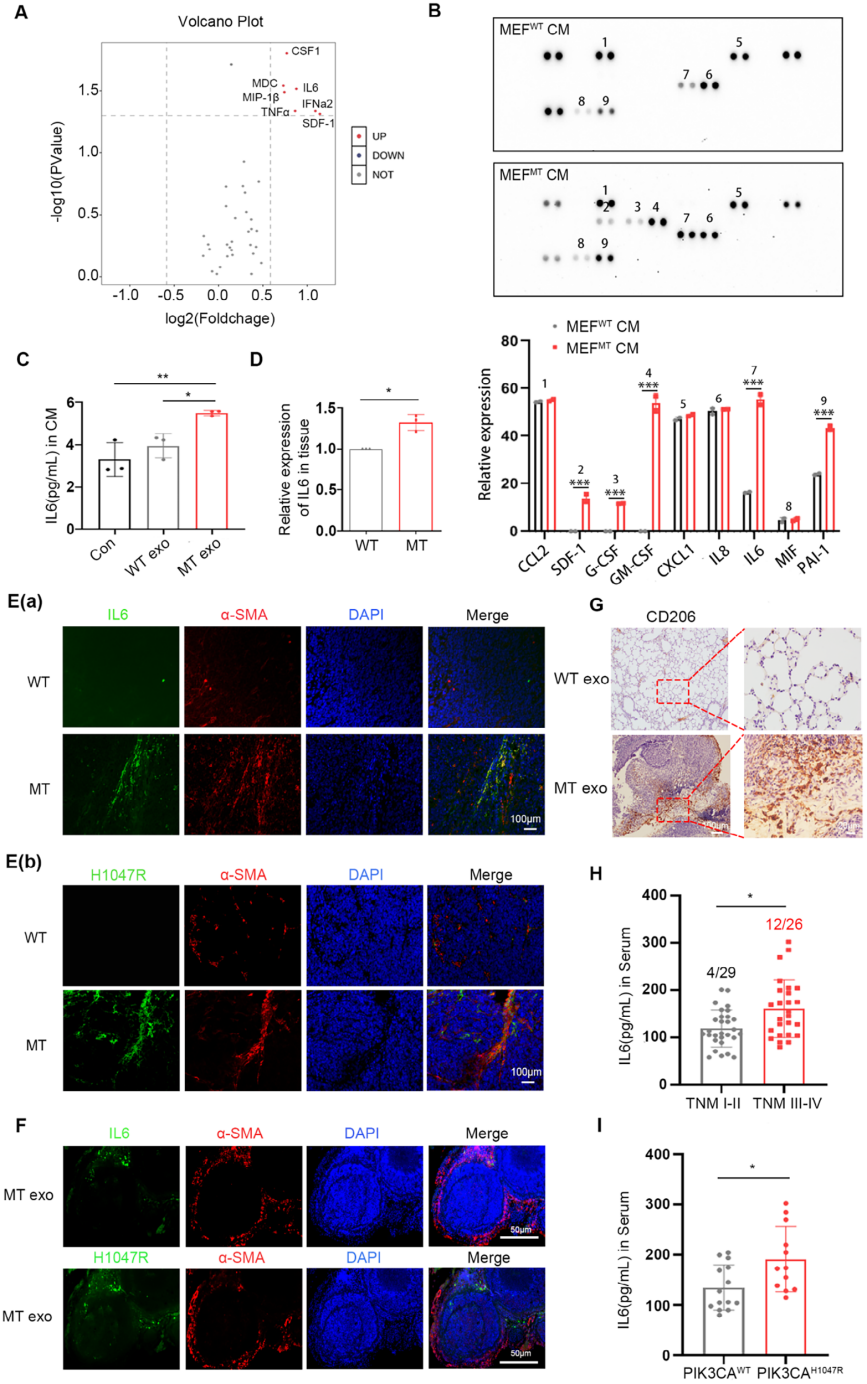
The horizontal transmission of exosomal *PIK3CA* ^H1047R^ mutation induces CAFs to secrete elevated levels of IL6. A) Olink targeted proteome detection the different MEFs CM. The specific cytokines marked in the upper right corner are those that were elevated in the MEFs^MT^ CM group. B) Cytokine Analysis Array System analysis of the different MEFs CM. Quantitative analysis is shown in the below panel. Error bars, mean±SD. Two‑way ANOVA with multiple comparisons. *** *P* < 0.0001, *n *= 2. C) ELISA analysis of IL6 expression in CM from MEFs treated with WT or MT cell‐derived exosomes. Error bars, mean±SD. One‑way ANOVA with multiple comparisons. * *P* < 0.01, ** *P* < 0.001, *n *= 3. D) Real‐time PCR analysis of IL6 expression in tumors from mice with WT and MT cells subcutaneously injection. Error bars, mean ± SD. Two‐sided Student's *t*‐test. * *P* < 0.01, *n *= 3. E) a,b) Immunofluorescence analysis of IL6, H1047R, and α‐SMA expression in paraffin sections of tumors from mice with WT and MT cells subcutaneously injection. Scale bar, 100 µm. F) Immunofluorescence analysis of IL6, H1047R, and α‐SMA expression in paraffin sections of lung tissue from mice with WT and MT cell‐derived exosomes tail vein injection. Scale bar, 50 µm. G) Immunohistochemical analysis of CD206 in paraffin sections of lung tissue from mice with WT and MT cell‐derived exosomes tail vein injection. Scale bar, 100 µm (left) and 20 µm (right). H) ELISA analysis of IL6 in serum of patients with CRC with different TNM stages. Error bars, mean ± SD. Two‐sided Student's t‐test. * *P* < 0.01, n = 55. I) Quantitative analysis of IL6 secretion from different *PIK3CA* mutation statuses in the serum from the TNM III‐IV patients. Error bars, mean ± SD. Two‐sided Student's t‐test. * *P* < 0.01, n = 26.

The primary role of CAFs is the production of paracrine cytokines, which in turn promote tumor metastasis.^[^ ^32^ ^]^ We hypothesized that the release of specific factors by CAFs facilitates CRC metastasis. To ascertain cytokine levels, we used Olink‐targeted proteome detection to identify CM from MEFs^WT^ and MEFs^MT^. Approximately forty cytokines were secreted, among which seven, including colony‐stimulating factor (CSF), recombinant macrophage‐derived chemokine (MDC), IL6, macrophage inflammatory protein‐1(MIP‐1), interferon, alpha 2 (IFNα2), tumor necrosis factor (TNF), and stromal cell‐derived factor‐1 (SDF‐1), exhibited significantly elevated protein levels in MEFs^MT^ CM (**Figure** **5**A). We also conducted secretion assays on CM from MEFs^WT^ and MEFs^MT^ using a Cytokine Analysis Array System, which enabled a comprehensive evaluation of the secretion of thirty‐six cytokines. The findings indicated that the levels of SDF‐1, granulocyte CSF (G‐CSF), granulocyte‐macrophage CSF (GM‐CSF), IL6, and plasminogen activator inhibitor‐1 (PAI‐1) in the MEFs^MT^ CM were significantly higher than those in MEFs^WT^ CM (Figure 5B). These results were further validated using the CM from WT and MT‐overexpressing MEFs, which yielded similar results (Figure S10A, Supporting Information). In both detection methods, the secretion levels of IL6 exhibited a pronounced elevation. Quantification of IL6 secretion in MEFs^WT^ and MEFs^MT^ CM was performed using ELISA, and the results were consistent with those of the above assays (Figure 5C). Validation of CM from WT and MT‐overexpressing MEFs yielded identical results (Figure S10B, Supporting Information).

To confirm the outcomes of the in vitro experiment, the expression of IL6 in the tumors of mice subcutaneously injected with WT and MT cells was assessed using real‐time PCR, revealing a substantial elevation in the MT group (Figure 5D). Subsequently, the expression of IL6 in tumors from mice subcutaneously injected with WT and MT cells was measured using ELISA, yielding results consistent with those obtained using real‐time PCR (Figure S10C, Supporting Information). Furthermore, immunofluorescence analysis of paraffin sections from mice subcutaneously injected with WT and MT cells demonstrated elevated IL6 levels in the MT group (Figure 5E(a)). Additionally, it was observed that IL6 exhibited co‐localization with H1047R and α‐SMA in serial sections (Figure 5E(b)). The serum of mice with subcutaneously injected with WT and MT cells was also analyzed using ELISA, and it was found that IL6 was highly expressed in the MT group (Figure S10D, Supporting Information). The *PIK3CA* ^H1047R^ mutation may induces CAFs to highly secret IL6 in primary tumors.

Tumor cell‐derived exosomes can also signal future metastatic sites over long distances to promote the formation of PMN for the targeted metastasis of disseminated tumor cells. To investigate the role of the exosomal *PIK3CA* ^H1047R^ mutation in the formation of the PMN prior to metastasis in distant organs, paraffin sections of lung metastatic tissues from the MT cell‐derived exosomes group were examined. IL6 exhibited co‐localization with H1047R and α‐SMA in serial sections (Figure 5F). Further examination of serum samples obtained from the orbital in the mouse tail vein metastasis model. ELISA results demonstrated a significant increase in IL6 expression in the MT cell‐derived exosomes group (Figure S10E, Supporting Information). Consequently, it can be inferred that the horizontal transfer of the *PIK3CA* ^H1047R^ mutation via exosomes derived from MT cells can induce fibroblast activation and the subsequent secretion of IL6 both locally and in the distant PMN, which serves as a crucial cytokine facilitating the interaction between CAFs and CRC cells. Macrophage activation plays a major role in the IL6‐activated PMN.^[^ ^33^ ^]^ We examined the expression of the macrophage activation marker CD206 in paraffin sections of lung tissues using immunohistochemistry and found that CD206 was highly expressed in metastatic foci (Figure 5G), suggesting that the highly secreted IL6 after fibroblast activation induced by the exosomal *PIK3CA* ^H1047R^ mutation in vivo might activate macrophages in the distal organs to promote the formation of PMN for targeted metastasis of disseminated tumor cells.

We further assessed the correlation between serum IL6, serum exosomal *PIK3CA* ^H1047R^ mutation, and metastatic capacity in patients with CRC. We evaluated the expression of IL6 in serum and the *PIK3CA* ^H1047R^ mutation in serum‐derived exosomes in fifty‐five patients with CRC, of whom twenty‐nine had TNM stages I–II and twenty‐six had TNM stages III–IV. Serum levels of IL6 expression were significantly elevated in TNM stage III–IV compared to those TNM stage I–II (Figure 5H). Further sequencing of serum‐derived exosomes from these patients revealed four *PIK3CA* ^H1047R^ mutations in the TNM stage I–II patients, whereas the *PIK3CA* ^H1047R^ mutation was present in twelve patients with TNM stage III–IV, as shown in Figure 5H. We also grouped patients with TNM stage III–IV with or without the *PIK3CA* ^H1047R^ mutation and assessed the expression of IL6 in each group. The expression level of IL6 was elevated in the *PIK3CA* ^H1047R^ mutation group (Figure 5I). We deduced that in patients with CRC, the expression of IL6 was associated with metastasis and correlated with the mutation frequency of exosomal *PIK3CA* ^H1047R^.

### 2.7 CAFs Promote CRC Metastasis through the PI3K/NF‐κB/IL6/ STAT3 Pathway

During the investigation of the correlation between IL6 and tumorigenesis, it has been documented that IL6 is subject to regulation by the nuclear transcription factor NF‐κB, and the p50/p65 heterodimer has been identified as the predominant binding site.^[^ ^34^ ^]^ Heightened NF‐κB activity can prompt cells to secrete elevated IL6 and other cytokines.^[^ ^35^, ^36^, ^37^ ^]^ Additionally, the activity of PI3K can regulate NF‐κB‐mediated signaling.^[^ ^38^ ^]^

**Figure 6:**
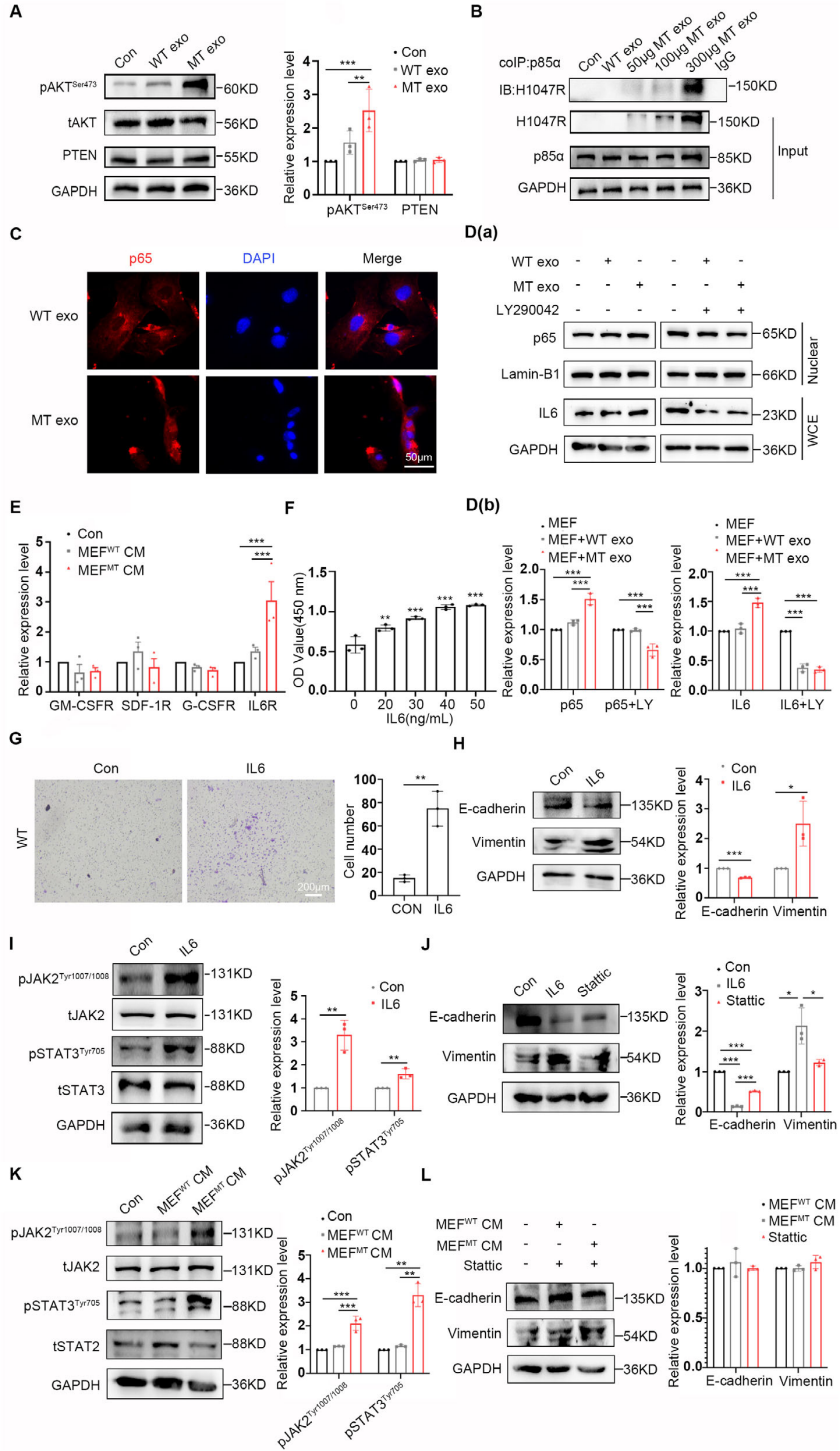
CAFs promote CRC metastasis through the IL6/JAK2/STAT3 pathway. A) Western blot analysis of PI3K signal expression in MEFs treated with WT or MT cell‐derived exosomes. Quantitative analysis is shown in the right panel. Error bars, mean ± SD. Two‑way ANOVA with multiple comparisons. ** *P* < 0.001, *** *P *< 0.0001, *n *= 3. B) co‐IP analysis was performed to examine the exogenous PIK3CA^H1047R^ competitive binding to p85α intracellularly in MEFs in an anexosome dose manner. C) Immunofluorescence analysis of p65 subcellular localization in MEFs treated with WT or MT cell‐derived exosomes. Scale bar, 50 µm. D) a) Western blotting analysis of P65 and IL6 expression of MEFs treated with WT or MT cell‐derived exosomes in the absence or presence of LY290042 (30 µm) pretreated for 24 h. D) b) Quantitative analysis of D(a). WCE, whole‐cell extract. Error bars, mean ± SD. Two‑way ANOVA with multiple comparisons. *** *P* < 0.0001, *n *= 3. E) Real‐time PCR analysis of GM‐CSFR, SDF‐1R, G‐CSFR, and IL6R expression in WT cells treated with different MEFs CM. Error bars, mean±SD. Two‑way ANOVA with multiple comparisons. *** *P* < 0.0001, *n *= 3. F) CCK8 assay to determine WT cells proliferation viability treated with exogenous IL6 in a dose manner. Error bars, mean ± SD. One‑way ANOVA with multiple comparisons. ** *P* < 0.001, *** *P* < 0.0001, *n *= 3. G) Transwell assay to determine the migration viability of WT cells treated with exogenous IL6 (40 ng mL^−2^). Representative images are shown. Quantitative analysis is shown in the right panel. Error bars, mean ± SD. Two‐sided Student's *t*‐test. ** *P *< 0.001, *n *= 3. Scale bar, 200 µm. H) Western blot analysis of EMT markers E‐cadherin and Vimentin in WT cells treated with exogenous IL‐6 (40 ng mL^−1^). Quantitative analysis is shown in the right panel. Error bars, mean ± SD. Two‑way ANOVA with multiple comparisons. * *P* < 0.01,*** *P* < 0.0001, *n *= 3. I) Western blot analysis of STAT3 signaling pathway of WT cells treated with exogenous IL6 (40 ng mL^−1^). Quantitative analysis is shown in the right panel. Error bars, mean ± SD. Two‑way ANOVA with multiple comparisons. ** *P* < 0.001, *n *= 3. J) Western blot analysis of EMT marker E‐cadherin and Vimentin of WT cells treated with IL6 (40 ng mL^−1^) in the absence or presence of Stattic (20 µm) pretreated for 8 h. Quantitative analysis is shown in the right panel. Error bars, mean±SD. Two‑way ANOVA with multiple comparisons. * *P* < 0.01, *** *P* < 0.0001, *n *= 3. K) Western blot analysis of STAT3 signaling pathway in WT cells treated with different MEFs CM. Quantitative analysis is shown in the right panel. Error bars, mean ± SD. Two‑way ANOVA with multiple comparisons. ** *P* < 0.001, *** *P* < 0.0001, *n *= 3. L) Western blot analysis of EMT marker E‐cadherin and Vimentin for WT cells treated with different MEFs CM, and Stattic (20 µm) pretreated for 8 h before MEFs CM was applied. Quantitative analysis is shown in the right panel. Error bars, mean ± SD. Two‑way ANOVA with multiple comparisons. *n *= 3.

Furthermore, we demonstrated that following the co‐incubation of MEFs with exosomes derived from MT cells for 72 h, the PI3K pathway in MEFs was activated, whereas PTEN remained unchanged (**Figure** **6**A). Notably the PI3K signaling pathway is also regulated by the tumor suppressor PTEN, and inactivation of PTEN results in constitutive activation of the PI3K pathway.^[^ ^39^ ^]^ Therefore, unchanged PTEN levels serve as a crucial requirement to demonstrate that increased downstream PI3K activity is indeed a result of the horizontal transmission of the exosomal *PIK3CA* ^H1047R^ mutation. To validate these findings, we conducted experiments using WT and MT‐overexpressing MEFs, which demonstrated that MT overexpression increased PI3K activity without affecting PTEN expression (Figure S11A, Supporting Information). The PIK3CA^H1047R^ catalytic subunit exerts its activity intracellularly by binding to the p85α regulatory subunit, therefore, we analyzed whether the exogenous PIK3CA^H1047R^ is dependent on p85α binding, which in turn induces PI3K activation. We observed that exogenous PIK3CA^H1047R^ competitive binding to p85α intracellularly in MEFs in an exosome dose‐dependent manner, as demonstrated using the co‐immunoprecipitation (co‐IP) assay (Figure 6B). This further demonstrated that the exosomal *PIK3CA* ^H1047R^ mutation caused functional changes. Replication of these experiments using WT and MT‐overexpressing MEFs yielded consistent results, further validating our findings (Figure S11B, Supporting Information).

It was hypothesized that the activation of fibroblasts was influenced by the involvement of PI3K/NF‐κB. Immunofluorescence analysis revealed that the co‐incubation of MEFs with exosomes derived from MT cells led to an increase in the nuclear localization of p65 (Figure 6C), thereby confirming the activation of the NF‐κB signaling pathway. Furthermore, the upregulation of IL6 was observed. Additionally, inhibition of the PI3K pathway was achieved by pretreatment with LY290042 for 24 h, resulting in a decrease in the expression of p65 and IL6 (Figure 6D(a,b)). These results indicate that the horizontal transmission of *PIK3CA* ^H1047R^ mutation via exosomes induces CAFs to secrete elevated levels of IL6 by activating the PI3K/NF‐κB signaling pathway.

Additionally, MEFs^WT^ and MEFs^MT^ CM were collected for co‐incubation with WT cells, and the receptor expression of SDF‐1 (SDF‐1R), G‐CSF (G‐CSFR), GM‐CSF (GM‐CSFR), and IL6 (IL6R) was detected using real‐time PCR. Only the expression of IL6R increased, as depicted in Figure 6E, thereby providing further evidence that IL6 functions by binding to IL6R in WT cells. To investigate the role of IL6 in CRC, WT cells were subjected to exogenous recombinant protein IL6 in a dose‐dependent manner, and the proliferation capacity of WT cells was assessed using the CCK8 assay. These findings demonstrate that exogenous IL6 increases WT cell proliferation in a dose‐dependent manner (Figure 6F). Notably, at 50 ng mL^−1^ of exogenous IL6, the proliferation capacity of WT cells reached a plateau. Consequently, 40 ng mL^−1^ IL6 was selected for subsequent experiments. Subsequently, WT cells were treated with exogenous IL6, which promoted their migration (Figure 6G) and EMT (Figure 6H) in WT cells. This finding suggests that IL6 has the potential to enhance CRC metastasis.

Previous research has indicated the significance of the STAT3 signaling pathway in mediating the downstream effects of IL6.^[^ ^40^ ^]^ Consequently, we assessed whether IL6‐induced CRC metastasis was dependent on the STAT3 signaling pathway. IL6 was administered to WT cells for 24 h. These findings demonstrated that IL6 led to increased expression of pJAK2 and pSTAT3 (Figure 6I). The STAT3 signaling pathway inhibitor, static, was also administered to WT cells for 8 h prior to IL6, resulting in the partial reversal of IL6‐induced EMT (Figure 6J). CM from MEFs^WT^ and MEFs^MT^ were also collected and co‐incubated with WT cells. These findings demonstrate that MEFs^MT^ CM increased the expression of pJAK2 and pSTAT3 (Figure 6K). When the inhibitor of the STAT3 signaling pathway, stattic, was introduced for 8 h before MEFs^MT^ CM was applied, the occurrence of EMT was partially reversed (Figure 6L). In conclusion, these findings provide evidence that CAFs facilitate CRC metastasis via the IL6/STAT3 pathway in vitro.

Through immunohistochemical staining of tissues from WT and MT cell tumor‐bearing mice, we demonstrated in vivo that the expression levels of pAKT and p65 in fibroblasts, as well as those of pJAK2 and pSTAT3 in tumor cells, were elevated in the MT cell‐bearing mouse group (Figure S12A,B, Supporting Information). This suggests that the horizontal transmission of the *PIK3CA* ^H1047R^ mutation via exosomes induces CAFs to secrete elevated levels of IL6 by activating the PI3K/NF‐κB signaling pathway. CAFs enhance CRC metastasis via the IL‐6/STAT3 signaling pathway in vivo.

**Figure 7:**
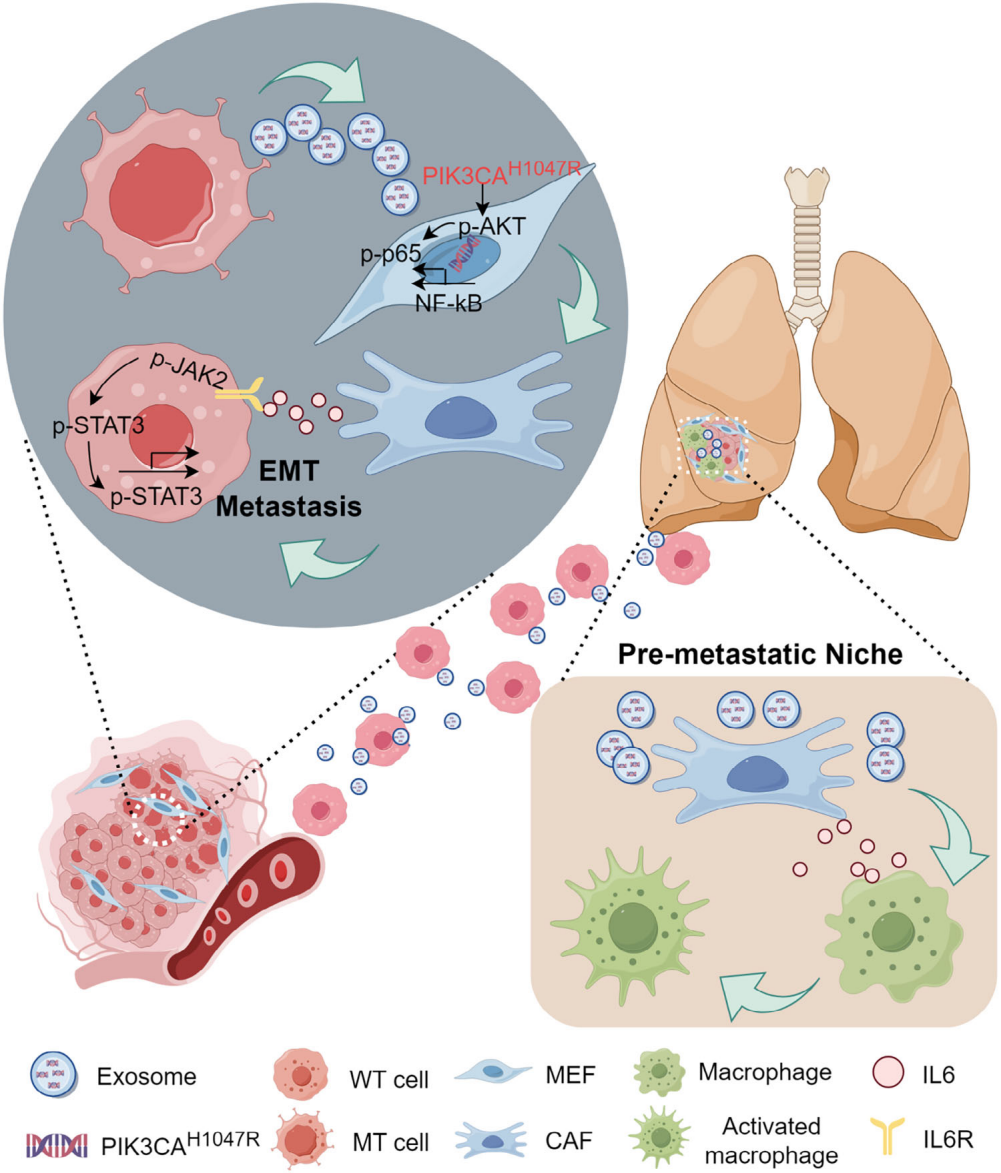
The schematic diagram of our findings. The *PIK3CA* ^H1047R^ mutation in MT‐cell‐derived exosomes activated the transformation of fibroblasts into CAFs through PI3K/NF‐κB, and the CAFs released a large amount of IL6, which promoted EMT by activating the STAT3 signaling pathway at the primary tumor site, and activation of macrophages in the distal organs, which led to the formation of an PMN leads to directional metastasis of tumor cells at the primary site.

A schematic diagram summarizes our findings (**Figure** **7**). Specifically, the presence of the *PIK3CA* ^H1047R^ mutation in MT‐cell‐derived exosomes activated the transformation of fibroblasts into CAFs through PI3K/NF‐κB, and the CAFs released a large amount of IL6, which promoted EMT by activating the STAT3 signaling pathway at the primary tumor site, and activation of macrophages in the distal organs, which led to the formation of a PMN leads to directional metastasis of tumor cells at the primary site. Additionally, we confirmed that the *PIK3CA* ^H1047R^ mutation in serum‐derived exosomes had a mutation frequency of 29.1% in patients with CRC, which was correlated with CRC metastasis. Patients with CRC with harboring the *PIK3CA* ^H1047R^ mutation in serum‐derived exosomes exhibited elevated levels of serum IL6 secretion, both of which are closely associated with CRC metastasis.

## 3 Discussion

Our study demonstrated that the metastasis of WT cells involves the horizontal transfer of the *PIK3CA* ^H1047R^ mutation in MT cell‐derived exosomes to fibroblasts, subsequently activating CAFs to release IL6, and promoting tumor cells' EMT in the primary foci. Additionally, our findings suggest that horizontal transfer of the *PIK3CA* ^H1047R^ mutation in MT cell‐derived exosomes to distal lung tissues may also occur, leading to the activation of fibroblasts and the subsequent release of IL6. This results in the activation of macrophages in the distal organs, forming PMN and enabling directional metastasis of tumor cells from the primary site.

Despite reports addressing the presence of DNA in exosomes, a notable degree of controversy persists regarding this subject. Various perspectives have been presented in previous studies. Some studies have posited the existence of DNA in large extracellular vesicles but not in exosomes,^[^ ^41^, ^42^ ^]^ whereas others have proposed that exosomal DNA is predominantly located on the surface of exosomes rather than internally.^[^ ^43^, ^44^ ^]^ Other studies have suggested that exosomal DNA is restricted to specific subpopulations, including CD63‐positive exosomes.^[^ ^45^, ^46^ ^]^ To confirm the presence of *PIK3CA* ^H1047R^ mutation DNA within exosomes, we isolated exosomes using classical ultracentrifugation. Subsequent NTA indicated that the majority of the exosomes were ≈100 nm in size. In the analysis of exosomal DNA, it is commonly accepted that the initial step involves the digestion of cell‐free DNA and non‐exosomal DNA outside the exosomes using DNase I and DNase I treatment was incorporated into our experimental protocols. DNA is restricted to CD63‐positive exosomes,^[^ ^45^, ^46^ ^]^ therefore, we employed a co‐incubation approach using magnetic beads coated with an antibody specific to the exosome marker, CD63. Subsequent DNA extraction from the co‐incubated exosomes revealed that the *PIK3CA* ^H1047R^ mutation was present in CD63‐positive exosomes derived from MT cells, providing further evidence that the extracted DNA was derived from exosomes rather than from other extracellular vesicles. In addition to exosome size and DNA isolation techniques, which can affect exosomal DNA detection, the selective packaging mechanism within exosomes may also play an important role. Lazaro‐Ibanez^[^ ^47^ ^]^ et al. demonstrated the presence of DNA in exosomes and showed that the DNA content differed across vesicle subtypes, suggesting a possible selective packing of DNA into various types of vesicles. Zhang^[^ ^48^ ^]^ et al. discovered that DNA was found in most tumor cell‐derived exosomes, however, its distribution differed between tumor cell lines. However, the precise mechanism through which DNA is packaged into exosomes remains unclear. Furthermore, we investigated the presence of DNA in exosomes derived from CRC cells other than LS174T, specifically LOVO, SW620, SW480, HT29, RKO, and HCT116 cells. HCT116, SW480, LS174T, and HT29 cell‐derived exosomes contained higher amounts of DNA than that in RKO and LOVO cell‐derived exosomes. This implies selective packaging of DNA within exosomes, potentially contributing to the varying conclusions found in different reports on exosomes containing DNA. The mutation frequency of *PIK3CA* ^H1047R^ in exosomes derived from the HCT116, RKO, and LS174T cell lines harboring the *PIK3CA* ^H1047R^ mutation was examined using ddPCR. We demonstrated that the exosomes derived from LS174T cells used in this study exhibited a higher mutation frequency of *PIK3CA* ^H1047R^ than exosomes derived from the other two cell lines. Although the total DNA content of HCT116 cells was comparable to that of LS174T cells, there was a disparity in the mutation frequency of *PIK3CA* ^H1047R^. This variability may be attributed to the selective encapsulation of distinct mutant genes by exosomes derived from diverse cell types. Notably, our study revealed varying quantities of *PIK3CA* ^H1047R^ mutant DNA carried by exosomes from different CRC cell origins, which is a novel finding in this field.

The primary role of exosomes is to facilitate intercellular communication by serving as delivery vehicles. However, the specific mechanism through which exosomal DNA contributes to this process remains unclear. Exosomal DNA enters the recipient cells and undergoes transcription and translation to exert its functional effects. Balaj^[^ ^20^ ^]^ et al. have documented that tumor exosomes harbor 5ʹ promoter regions, 3ʹ untranslated regions, and active reverse transcriptional transposons. These exosomal components can be transferred between cells through endocytosis or fusion, and exosomal *c‐Myc* is transferred to recipient cells, where they subsequently undergo transcription and translation. Cai^[^ ^49^ ^]^ et al. demonstrated that K562 cell‐derived exosomal angiotensin II type 1 DNA can be transfered to HEK293 cells and affect Na^+^–K^+^ ATPase activity. Our research demonstrated that exosomes derived from MT cells can transfer the *PIK3CA* ^H1047R^ mutation to fibroblasts, where it can be transcribed and translated, shedding light on the mechanism by which *PIK3CA* ^H1047R^ mutation enters recipient cells. To exclude the effects of exosomal RNA and protein delivery, we co‐incubated MEFs with MT cell‐derived exosomes and the transcriptional inhibitor ActD. The H1047R expression level was reduced by ≈75% compared with that in the non‐ActD‐treated group, suggesting that H1047R expression in MEFs was predominantly due to exosomal *PIK3CA* ^H1047R^ mutation DNA delivery, which plays an important role. Additionally, the PIK3CA^H1047R^ mutation relies on its interaction with p85α.^[^ ^50^ ^]^ Our study demonstrated that the functionality of the PIK3CA^H1047R^ mutation in MEFs was dependent on its binding to p85α. DNA exists stably in the recipient cells. Lee^[^ ^18^ ^]^ et al. demonstrated that *H‐ras* signals are still present in passaged recipient cells for as long as 30 days. Domenis^[^ ^51^ ^]^ et al. found that mutated *TP53* in passaged recipient cells for up to 10 days without exosome treatment, suggesting its possible integration into their genome. Moreover, our study indicated that the presence of the *PIK3CA* ^H1047R^ mutation in the genome of fibroblasts. The *PIK3CA* ^H1047R^ mutation was also identified in mCAFs through LCM of paraffin sections of MT cell tumor‐bearing mice, suggesting the amplification of cells containing this mutation in vivo. Subsequently, mCAFs were isolated from the tumors of WT and MT tumor‐bearing mice, and Sanger sequencing of fifth‐passage mCAFs confirmed the presence of the *PIK3CA* ^H1047R^ mutation. Our findings provide new evidence and enrich existing research.

Research on mutations in patients with tumors has primarily focused on the tumor cells themselves, with less emphasis on stromal cells. However, as the significance of TME in tumorigenesis has been extensively investigated, it has become evident that stromal cells harbor mutations and play a crucial role in tumor development and progression. The integration of single‐cell RNA sequencing, whole genome sequencing, and exome sequencing,^[^ ^52^ ^]^ as well as multiplex single‐cell RNA sequencing and multiplex single‐cell MALBAC genome sequencing, could identified evidence of gene mutations in the CAFs of CRC and found that *BGN*, *RCN3*, *TAGLN*, *MYL9*, and *TPM2* mutations are prevalent in CRC, which plays a crucial role in the pathogenesis and progression of CRC.^[^ ^53^ ^]^ However, these reports did not specify the presence of *PIK3CA* ^H1047R^ mutation, and the origin and function of these mutations in CAFs remain unclear. Our findings showed that the CRC cell‐derived exosomal *PIK3CA* ^H1047R^ mutation could be horizontally transmitted to fibroblasts. The *PIK3CA* ^H1047R^ mutation was also detected in hCAFs of patients with CRC, indicating the presence of mutations in stromal cells, which is consistent with current reports. Moreover, whether the *PIK3CA* ^H1047R^ mutation is present in CAFs has not yet been reported. In this study, we observed the delivery of CRC cell‐derived exosomal *PIK3CA* ^H1047R^ to MEFs and hfbs, suggesting the possibility of transferring exosomal *PIK3CA* ^H1047R^ mutations in vitro. Our identification of the *PIK3CA* ^H1047R^ mutation in mCAFs from MT cell tumor‐bearing mice further supports the potential for CRC cell‐derived exosomal *PIK3CA* ^H1047R^ mutation transfer in vivo. Collectively, these findings indicate that fibroblasts can acquire mutations from tumor cells both in vitro and in vivo, providing new insights into the impact of stromal cell mutations on tumorigenesis.

An increasing number of studies have identified the diagnostic significance of mutation in the serum exosomes of patients with tumors. Castellanos‐Rizaldos^[^ ^54^ ^]^ et al. observed that exosomal DNA for *EGFR* ^T790M^ detection has elevated sensitivity and specificity in patients with non–small‐cell lung cancer, and Yang^[^ ^13^ ^]^ et al. demonstrated the potential clinical utility of exosomal DNA for the identification of *KRAS* ^G12D^ and *TP53* ^R273H^ mutations in patients with pancreas‐associated pathologies. In this study, we examined serum‐derived exosomes from patients with CRC and detected the presence of an exosomal *PIK3CA* ^H1047R^ mutation, consistent with previous findings.^[^ ^26^, ^55^ ^]^ Statistical analysis revealed a significant correlation between exosomal *PIK3CA* ^H1047R^ mutations and metastasis in patients with CRC. Furthermore, we observed elevated levels of serum IL6 secretion in patients with CRC, especially in those harboring the *PIK3CA* ^H1047R^ mutation, which was significantly correlated with CRC metastasis. Consequently, the simultaneous identification of exosomal *PIK3CA* ^H1047R^ mutations and serum IL6 secretion can improve the accuracy of CRC diagnosis. Through mechanistic investigation and serological analysis, our study is the first to establish this correlation, offering a novel approach for non‐invasive detection.

Given the significance of the *PIK3CA* ^H1047R^ mutation in tumor development and advancement, ongoing studies are focusing on targeting this mutation as a potential therapeutic intervention.^[^ ^56^, ^57^ ^]^ Our findings demonstrated that the exosomal *PIK3CA* ^H1047R^ mutation enhances CRC metastasis by stimulating fibroblasts. Targeting specific CAFs or releasing specific exosomes to disrupt the selective packaging or delivery of *PIK3CA* ^H1047R^ may offer a novel approach for CRC. Although numerous studies have investigated the targeting of IL6 for tumor therapy,^[^ ^58^, ^59^ ^]^ the concurrent targeting of the *PIK3CA* ^H1047R^ mutation and IL6 has not been documented. Therefore, the precise simultaneous targeting of the *PIK3CA* ^H1047R^ mutation and IL6 is crucial to guide personalized therapeutic strategies.

## 4 Experimental Section

### CRC Patient Tissue Sample, Blood Sample

The tumor tissue and blood samples from patients with CRC were collected from Shengjing Hospital, China Medical University. The study was ethically approved by the Ethics Committee of China Medical University and written consent was obtained from all patients for the use of samples for study purposes before the procedure (Ethical code is [2023] 022). After blood samples were collected, serum was isolated at 4 °C by centrifugation at 3400 g for 10 m and these serum samples were frozen at −80 °C for subsequent use. The characteristics including age, tumor differentiation, sex, TNM stage, and lymph node metastasis were collected from patients with CRC.

### Primary Isolation of CAFs

The tumor tissues of xenografts model mice or the patients with CRC were taken and immediately rinsed with PBS. The tissues were then digested with 1 mg mL^−1^ of collagenase type I (Solarbio, China) overnight at 4 °C and then neutralize with CAF medium. CAFs were passaged at 1:3–1:4, and after each passage, the CAFs were attached for 10 m and the supernatant was discarded. After the CAFs were passed to the fifth generation, subsequent experiments were conducted.

### Cell Lines and Culture

The MT cell line was purchased from the Shanghai Cell Bank of the Chinese Academy of Sciences and the WT cell line was established by Yuanjing Biology Co., Ltd. (Ubigene, China). The tumor cell lines were cultured in RPMI 1640 medium (Corning, US) with 10% FBS (Sangon, China) supplemented with 100 U mL^−1^ Penicillin‐Streptomycin (PS). MEFs and CAFs were cultured in DMEM (Corning, US) with 10% FBS (Sangon, China) supplemented with 2 mm *L*‐glutamine (Invitrogen, USA), 100 U mL^−1^ PS in incubators at 37 °C, and 5% CO_2_.

### CM Preparation

The MEFs were continuously treated with 300 µg WT and MT cell‐derived exosomes for 72 h and then cultured in a complete medium for another 72 h. The CM was collected, centrifuged at 300 g for 10 m, 2000 g for 10 m, and 10 000 g for 30 m at 4 °C, filtered through a 0.22 µm filter membrane (Millipore, USA), and the samples were frozen at −80 °C for subsequent use.

### CCK8

The proliferative capacity of cells was measured using the CCK‐8 kit (Beyotime, China) according to the manufacturer's instructions. For the detection of MEFs, cells were seeded in 96‐well plates and treated with 300 µg WT and MT cell‐derived exosomes for 72 consecutive hours. To test the proliferation ability of tumor cells, WT cells were seeded in 96‐well plates and treated with different MEFs CM for 72 consecutive hours, or treated with IL6 in a dose‐manner for 24 h. The absorbance was measured using a microplate reader at dual wavelengths of 450 nm.

### Transwell and Scratch Assay

For the detection of MEF migration, cells were seeded in 6‐well plates and treated with 300 µg WT and MT cell‐derived exosomes for 72 consecutive hours. To evaluate the ability of WT cells, different MEFs CM or IL6 (40 ng mL^−1^) were applied to WT cells for 72 or 24 h. The cells were digested and adjusted to 5 × 10^4^ cells/100 µL by FBS‐free culture medium seeded into the upper chamber. A complete culture medium (600 µL) was added into the lower chamber. After 24 h, the well plates were stained with crystal violet (Beyotime, China). Image J was used to measure the number of cells passing through the chamber.

The wound healing assay was used to evaluate the migration of WT and MT cells. Cells grew to 80–90% confluency in 6‐well plates and formed wounds by dragging the plastic pipette tip across the cell surface. The remaining cells were washed three times in PBS to remove cell debris and incubated at 37 °C with an FBS‐free medium. The migrated cells at the front of the wound were photographed 24‐72 h later. Image J software was used to measure the wound area.

### Western Blot

The cells were dissolved in RIPA cell lysis buffer (Beyotime, China) with PMSF for 30 m on ice. The supernatant was collected by centrifugation. Subsequently, the supernatant was denatured using a 5 × loading buffer. It was then separated through by SDS‐PAGE, transferring the protein to the PVDF membrane (Millipore, USA) and blocking in TBST solution with 5% skim milk. The primary antibody was incubated overnight at 4 °C, and then the membrane incubated with horseradish peroxidase‐bound secondary antibody at room temperature for 1 h. Finally, the membranes were detected with an electroluminescence reagent (Beyotime, China).

### Isolation and Internalization of Extracellular Vesicles

Cells were cultured in complete media depleted of exosomes for 48 h and extracellular vesicles were isolated from supernatants. After the same centrifugation and filtration steps as detailed in the CM preparation, the supernatant was collected and the exosome was collected by centrifugation in an ultracentrifuge (Hitachi, Japan) at 200 000 g for 3 h at 4 °C. After the supernatant obtained by ultracentrifugation was discarded, 300 µL PBS was added, and resuspend to collect exosomes.

Exosome internalization was achieved by incubating MEF cells with DiD (Invitrogen, USA)‐labeled WT and MT cell‐derived exosomes for 12 h. The control group was incubated with PBS. MEFs were then stained with DAPI (Beyotime, China). Representative images were captured using a Zeiss microsystem.

To observe the exosome DNA transferred to the recipient cells, WT and MT cell‐derived exosomes were stained with 8 µg mL^−1^ AO (Solarbio, China) and then stained with DAPI (Beyotime, China), and the representative images about co‐localization of AO and the nucleus were captured using a Zeiss super‐resolution confocal microscopy.

### Transmission Electron Microscope

The exosomes were fixed in 2.5% glutaraldehyde for 12 h. Subsequently, the samples were sent to an electron microscopy center of China Medical University where the remaining steps were performed. Finally, the morphology of exosomes was observed under the transmission electron microscope.

### Isolation of DNA, PCR, and Sanger Sequencing

Genomic DNA kits (Tiangen, China) were used to extract DNA from cells and exosomes as recommended by the manufacturer. For the DNA extraction of exosomes, DNA on the surface of exosomes had to be removed first. After 8 µL DNase I (Takara, Japan) was added into 200 µL exosomes and digested at 37 °C for 30 m, then 25 µL 0.5 m EDTA (Sangon, China) was added and heat‐treated at 80 °C for 2 m to inactivate the enzyme, followed by the extraction of exosomes DNA. Subsequently, PCR amplification was performed for analysis of the PI*K3CA* ^H1047R^ mutation status.

A 126 bp fragment of the *PIK3CA* containing the H1047R mutation using PCR design primers: forward, 5'‐GGAGTATTTCATGAAAATGAATGATGCG‐3', and reverse, 5'‐GAGTTTCATTTTCGTSTATCTT‐3'. The PCR reaction was conducted under the following cycle conditions: 94 °C for 45 s, 60 °C for 45 s, and 72 °C for 45 s, with 30 cycles. The predicted PCR product size was 126 bp and confirmed by electrophoresis in a 3% agarose gel containing ethidium bromide.

The PCR products were sent to Sangon Biotech Co., Ltd. to detect the *PIK3CA* ^H1047R^ mutation status by direct sequencing. For samples that could not be detected by direct sequencing, the T‐A cloning was then used for sequencing.

### WES

Fibroblasts were treated with MT cell‐derived exosomes. After three generations, isolation of the fibroblast DNA and then the DNA products were sent to Sangon Biotech Co., Ltd. to detect the *PIK3CA* ^H1047R^ mutation status by WES.

### Plasmid Construction and Transfection

Plasmids encoding mouse PIK3CA^WT^ and PIK3CA^H1047R^ were constructed by Fenghui Corporation (Fenghbio, China). Opti‐MEM (250 µL) was mixed with 2.5 µg plasmid and 5 µL of p3000 (Invitrogen, USA), another 250 µL Opti‐MEM mixed with 3.75 µL lipo3000 (Invitrogen, USA). The two groups were gently blown and mixed, then allowed to stand for 20 m at room temperature. The medium without PS was replaced in the well plate before transfection. The mixed solution was then added to the 6‐well plate. Shake gently until evenly distributed. The protein and RNA were extracted after 48 h for subsequent analysis.

### Olink Targeted Proteome Detection

MEFs were cultured in a 6‐well plate. Upon reaching ≈70% confluency on the second day, exosomes derived from WT and MT cells were administered for consecutive 72 h. Subsequently, the CM from various groups were collected following a 72 h incubation with serum‐free medium. These CM were then submitted to APPLIED PROTEIN TECHNOLOGY Biotechnology Co., Ltd. for targeted proteomic analysis.

### RT‑PCR and Real‑Time PCR

Total RNA was isolated from cells using Trizol (Invitrogen, USA) according to the manufacturer's protocol. cDNA was obtained by reverse transcription from 1000 ng RNA using an RT‐PCR kit (TransGen, China). Realtime PCR was performed using SYBR (TransGen, China) according to the manufacturer's protocol. mGAPDH primers: forward, 5' ACCACAGTCCATGCCATCAC‐3', reverse,5'‐ TCCACCACCCTGTTGCTGTA ‐3'. mIL6 primers: forward, 5'‐ CTCCCAACAGACCTGTCTATAC‐3', reverse,5'‐ CCATTGCACAACTCTTTTCTCA‐3'; mMMP2 primers: forward, 5'‐ ACTTTGAGAAGGATGGCAAGTA‐3', reverse, 5'‐CTTCTTATCCCGGTCATAGTCC‐3'; mTGFβ primer: forward, 5'‐ ACCGCAACAACGCCATCTATGAG‐3', reverse, 5'‐ GGCACTGCTTCCCGAATGTCTG‐3'; mSDF‐1 primer: forward, 5'‐TCTGAAAATCCTCAACACTCCA‐3', reverse, 5'‐CAGGTACTCTTGGATCCACTTT‐3'; hIL6R primers: forward, 5'‐GCAAGTTCAGCAAAACTCAAAC‐3', reverse, 5'‐TTGTGAATGTCTTTGACCGTTC‐3'; hG‐CSFR primers: forward, 5'‐CCACCAACAGTACAGTCCTCACC‐3', reverse, 5'‐GAGCCAGGCAGTTCCACAGAG‐3'; hSDF‐1R primers: forward, 5'‐TGTCATCTACACAGTCAACCTC‐3', reverse, 5'‐CAACATAGACCACCTTTTCAGC‐3'; hGM‐CSFR primers: forward, 5'‐GCATCGTCCTCGGCTTCCTC‐3', reverse, 5'‐CCAGATGATCTCGTCTTCCACCTC ‐3'; hGAPDH primers: forward, 5' GGTCACCAGGGCTGCTTTTA‐3', reverse, 5'‐GGATCTCGCTCCTGGAAGATG‐3'. The 2^−ΔΔCt^ method was used to analyze the relative expression of each mRNA.

### ActD

ActD group was co‐treated with 80 nm ActD and 100 µg MT cell‐derived exosomes for 72 h, then cell lysates were collected.

### Immunofluorescence

The cells were washed with ice‐cold PBS and fixed for 30 m at room temperature with 4% paraformaldehyde. The cells were then permeabilized with 0.1% Triton X‐100 at room temperature for 5 m, blocked at room temperature in 5% BSA in PBS for 1 h, and the primary antibodies were added at 4 °C overnight. It was washed three times the next day with PBS, followed by Alexa Fluor‐conjugated secondary antibody for 1 h at room temperature, and washed three times again with PBS before staining in the dark with DAPI for 5 m. For the paraffin section, the immunohistochemical hypersensitivity UltraSensitiveTM SP kit (Maxim, China) was used for day 1 as recommended by the manufacturer. Day 2 was treated as the second day of the cell experiment.

### HE Staining

HE staining kits were purchased from the Beijing Solarbio Biotechnology Co., LTD. Tumor tissues from mice were fixed and embedded in paraffin, and HE staining analyses were performed as recommended by the manufacturer. Representative images were captured using a Nikon Eclipse Ni microsystem.

### Immunohistochemistry

Immunohistochemical hypersensitivity UltraSensitiveTM SP kits were purchased from the Maixin Biotechnology Co., LTD. Tumor tissues from mice were fixed and embedded in paraffin, and immunohistochemistryanalyses were performed as recommended by the manufacturer. Representative images were captured using a Nikon Eclipse Ni microsystem.

### Co‐IP

MEFs were seeded in 10 cm^2^ dishes, treated with 300 µg WT and MT cell‐derived exosomes for 72 consecutive h, or overexpressing PIK3CA^WT^ and PIK3CA^H1047R^. NP40 cell lysis buffer (500 µL) was added to cells for 30 m ice. Primary antibodies and protein A/G beads (Bimake, USA) were added to the cell lysate supernatant and rotated at 4 °C overnight. Then boiled in a 1 × SDS‐PAGE loading buffer and used for Western blot.

### Cytokine Array Experiment

CM of different MEFs were harvested and a Cytokine Analysis Array System, enabling the comprehensive evaluation of 36 cytokine secretions (R&D, USA) was evaluated according to the manufacturer's instructions.

### ELISA

For cell supernatants, CM of different MEFs was harvested. For animal serum, blood was taken from the mouse eye socket, allowed to stand at room temperature for 10 m, centrifuged at 1000 g at 4 °C for 5 m, the supernatant was taken. For the xenografts model mice, the tissue was collected in PBS. IL6 expression was quantified by ELISA according to the manufacturer's instructions (Biorbyt, China). The absorbance values of each well were measured with a microplate reader at dual wavelengths of 450 nm.

### Animal Experiment

The animal study protocol was approved by the Administrative Committee on Animal Research in China Medical University (the protocol code is KT20240911). To exclude individual differences in the establishment of the xenotransplantation model, 4 × 10^6^ WT and MT tumor cells into 200 µL PBS were injected into the left and right sides of the BALB/c female mice, respectively. Tumor growth was evaluated by measuring tumor length (*L*) and width (*W*) every other day and measured according to the formula (tumor volume = (*L* × *W* ^2^)/2). The mice were euthanized 14 days later, weighed and imaged. The expression of Ki67, FAP, α‐SMA, and H1047R were detected by immunohistochemistry.

In the tail intravenous injection metastasis model, to evaluate the WT and WT cells metastasis ability, 4×10^6^ tumor cells into 200 µL PBS were injected into the BALB/c female mice tail vein. To evaluate the correlation between *PIK3CA* ^H1047R^ mutation in exosomes and CRC metastasis, mice were divided into three groups: 1) PBS, 2) WT exosomes, 3) MT exosomes injected twice a week via the tail vein. A total of 4 × 10^6^ WT cells were then intravenously injected into BALB/c mice pretreated with exosomes/PBS. Continue to inject exosomes/PBS into the tail vein twice per week. Organs were taken for observation after 60 days. The expressions of H1047R and IL6 were detected by immunofluorescence.

### Collagen Gel Contraction

Cell contraction measurements were performed to evaluate the contractility of MEFs. MEFs were embedded in 100 µL collagen type I (Solarbio, China). The final cell density was 3 × 10^5^ cells mL^−1^ and the collagen content was 1 mg mL^−1^. The mixture was inoculated into 96‐well plates, incubated for 24 h in a humidified incubator at 37 °C and imaged. To calculate the relative collagen area (%), the images were quantified using Image J software.

### LCM

Paraffin sections were placed on Leica vinyl acetate film and microdissected on a Leica LMD6000 laser fiber cutting instrument. The continuous sections of α‐SMA positive immunohistochemistry were used as the control. The target cells were found through the observation on the computer screen. The α‐SMA positive cells were cut to the middle as far as possible to avoid the pollution of other cells at the edge. Due to the action of gravity, the separated cells fall into the pre‐assembled 200 µL PCR tube cap on the collector stage. The DNA was then extracted from the separated cells for further sequencing.

### Statistical Analysis

All data were analyzed with GraphPad Prism 8.0 (La Jolla, USA) and were shown as the means ± standard deviation (SD). Each experiment was carried out in technical and biological triplicate. The Student's *t*‐test two‐sides was used to compare two groups. One‐way ANOVA and two‐way ANOVA were used to determine multiple group comparisons. Fisher precise test was used to analyze the correlation of serum exosome mutation rate. A value of *P* < 0.05 was deemed statistically significant.

### Antibody

The antibodies used in this research are shown in Table S2 (Supporting Information).

## Conflict of Interest

The authors declare no conflict of interest.

## Supporting information



Supporting Information

## Data Availability

Research data are not shared.
