# Supplementary material for: Colorectal Cancer Cells–Derived Exosomal PIK3CA Mutation DNA Promotes Tumor Metastasis by Activating Fibroblast and Affecting Tumor Metastatic Microenvironment
Source: Adv Sci (Weinh). 2025 May 8;12(27):2501792. doi: 10.1002/advs.202501792 (PMC12279234; doi:10.1002/advs.202501792)
Supplement: Supplementary file 1 — Supporting Information [file ADVS-12-2501792-s001.docx]

Supporting Information

Colorectal Cancer Cells–Derived Exosomal *PIK3CA* Mutation DNA Promotes Tumor Metastasis by Activating Fibroblast and Affecting Tumor Metastatic Microenvironment

Rui Wang, Wanming Li, Yuqiong Lv, Wei Ba, Ying Jiang, Xiaoshuai Li, Jin Fang*


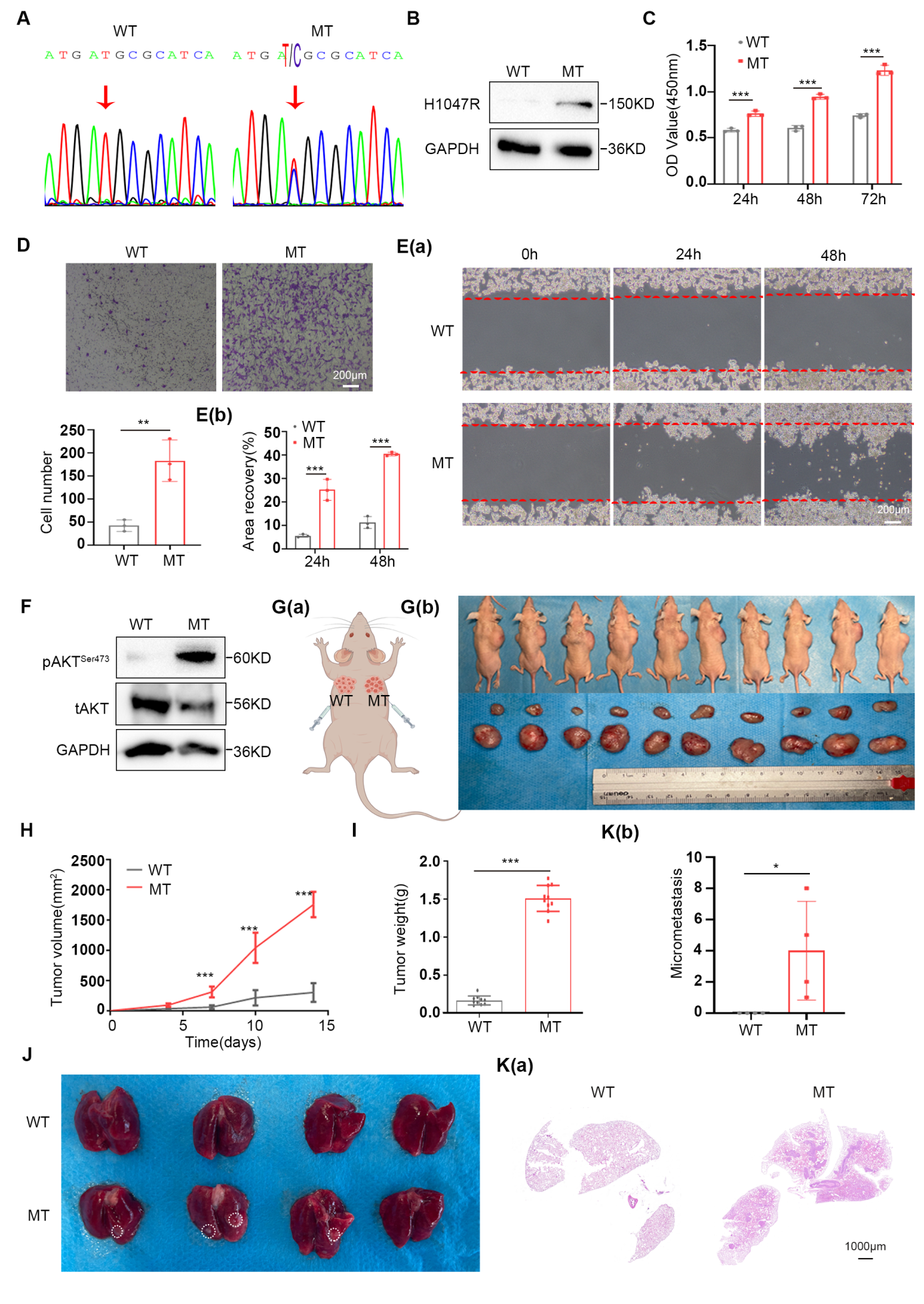


**Figure S1.** **LS174T cells with *PIK3CA*^H1047R^ mutation were gene**-**edited to *PIK3CA*^WT^ and functionally characterized.** A) Sanger sequencing identity of the mutation sites following gene editing in WT cells, MT cells were as a control. B) Western blot identity of the mutation sites following gene editing in WT cells. MT cells were as a control. C) CCK8 assay to determine the proliferation viability of WT and MT cells. Error bars, mean±SD. Two-way ANOVA with multiple comparisons. *** *P* < 0.0001, *n*=3. D) Transwell assay to determine the migration viability of WT and MT cells. Quantitative analysis is shown in the below panel. Error bars, mean±SD. Two-sided Student’s t test. ** *P* < 0.001, *n*=3. Scale bar, 200 μm. E) a) The relative width of the scratch assay in WT and MT cells. E) b) Quantitative analysis of E(a). Error bars, mean±SD. Two-way ANOVA with multiple comparisons. *** *P* < 0.0001, *n*=3. Scale bar, 200 μm. F) Western blotting analysis of PI3K activity levels in WT and MT cells. G) a) Schematic of the animal experimental design. G) b) Mice were euthanized 15 days after WT and MT cells were subcutaneously injecting into the left and right sides of BALB/c mice, gross observations of mice and the tumor tissues. *n*=10. H) The sizes of tumors were measured. Error bars, mean±SD. Two-way ANOVA with multiple comparisons. *** *P* < 0.0001, *n*=10. I) The tumor volumes were calculated. Error bars, mean±SD. Two-sided Student’s t test. *** *P* < 0.0001, *n*=10. J) Mice were euthanized 60 days after WT and MT cells (4×10^6^ cells in 200 μL PBS) tail intravenously injection, gross observations of the lung tissues. White circles are the metastatic foci in the lungs. *n*=4. K) a) HE staining evaluated the metastatic capability. K) b) Quantitative analysis of K(a). Error bars, mean±SD. Two-sided Student’s t test. *** *P* < 0.0001, *n*=4. Scale bar, 1000 μm.


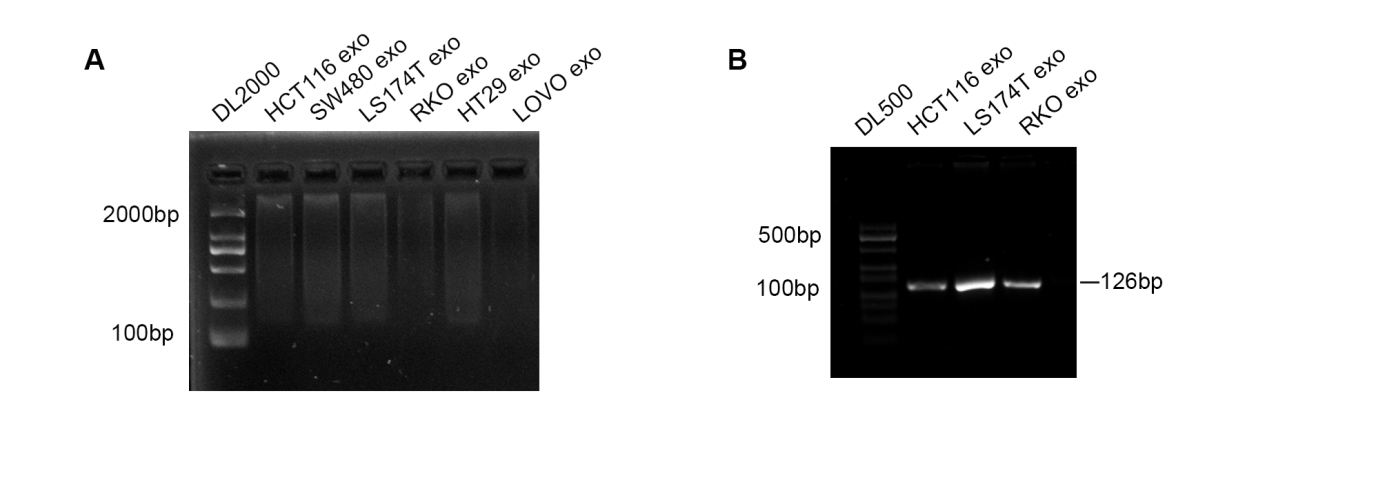


**Figure S2. DNA content in exosomes of different CRC cells was different.** A) DNAs in different CRC cell-derived exosomes on 1% agarose gel precasted with ethidium bromide. DNA ladder, DL2000. B) *PIK3CA* in exosomes from *PIK3CA*^H1047R^ mutation cells was detected by PCR, and the PCR products were analyzed on 3% agarose gel precasted with ethidium bromide. DNA ladder, DL2000.


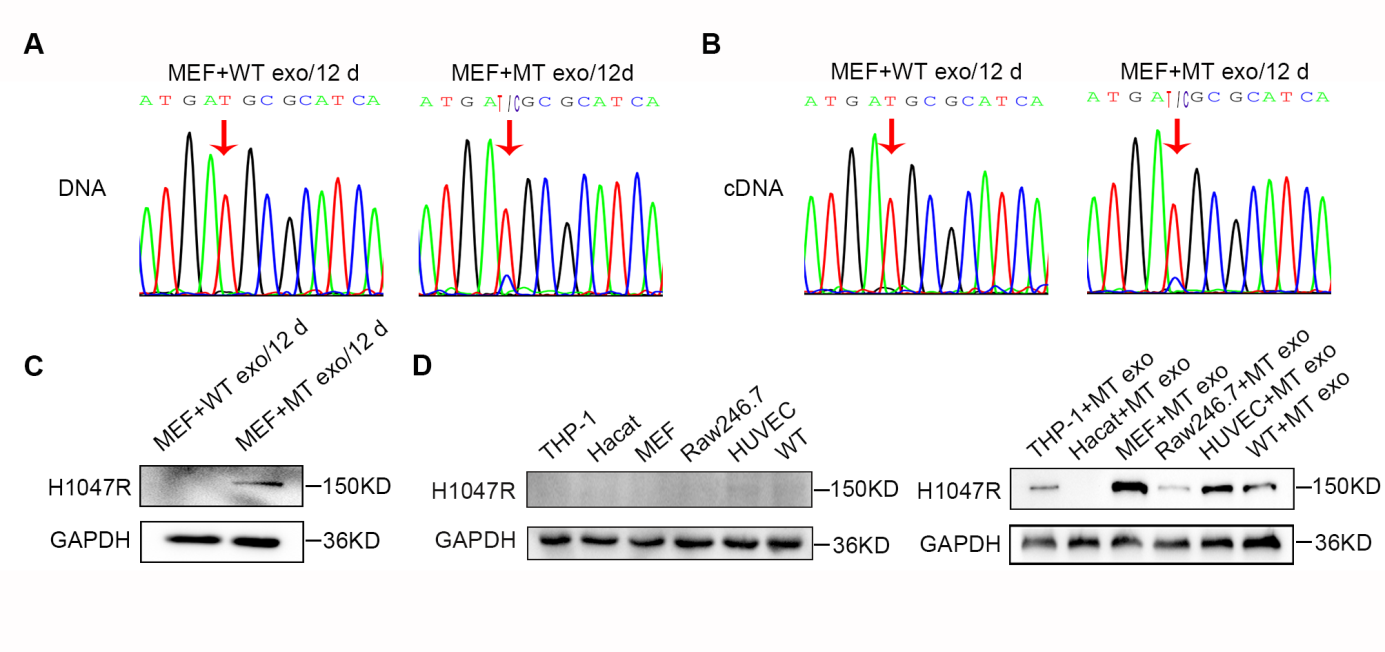


**Figure S3. Exosomal *PIK3CA*^H1047R^ mutation can be stable existed in MEFs.** A) Sanger sequencing analysis of MEFs DNA co-incubated with WT and MT cell-derived exosomes for 72 h, and then cultured for an additional 12 days without exosomes, the presence of *PIK3CA*^H1047R^ mutation in MEFs. B) Sanger sequencing analysis MEFs cDNA co-incubated with WT and MT cell-derived exosomes for 72 h, and then cultured for an additional 12 days without exosomes, the presence of *PIK3CA*^H1047R^ mutation in MEFs. C) Western blot analysis of H1047R expression in MEFs co-incubated with WT and MT cell-derived exosomes for 72 h, and then cultured for an additional 12 days without exosomes. D) Western blot analysis of H1047R expression after MT cell-derived exosomes act on THP-1, HaCaT, MEFs, Raw264.7 cells, HUVECs, and WT cells.

**Table S1.** WES analyses the sequences integrated into the recipient fibroblasts

| Chromosome | Region | Variation type | | Insert sequence | Geno  type | Mutation frequency [%] |
| --- | --- | --- | --- | --- | --- | --- |
| 17 | Exon | Indel | GAT...GCACGT...TGA^a)^ | | A/G | 11.07 |

^a)^ The base marked red is the H1047R mutation site


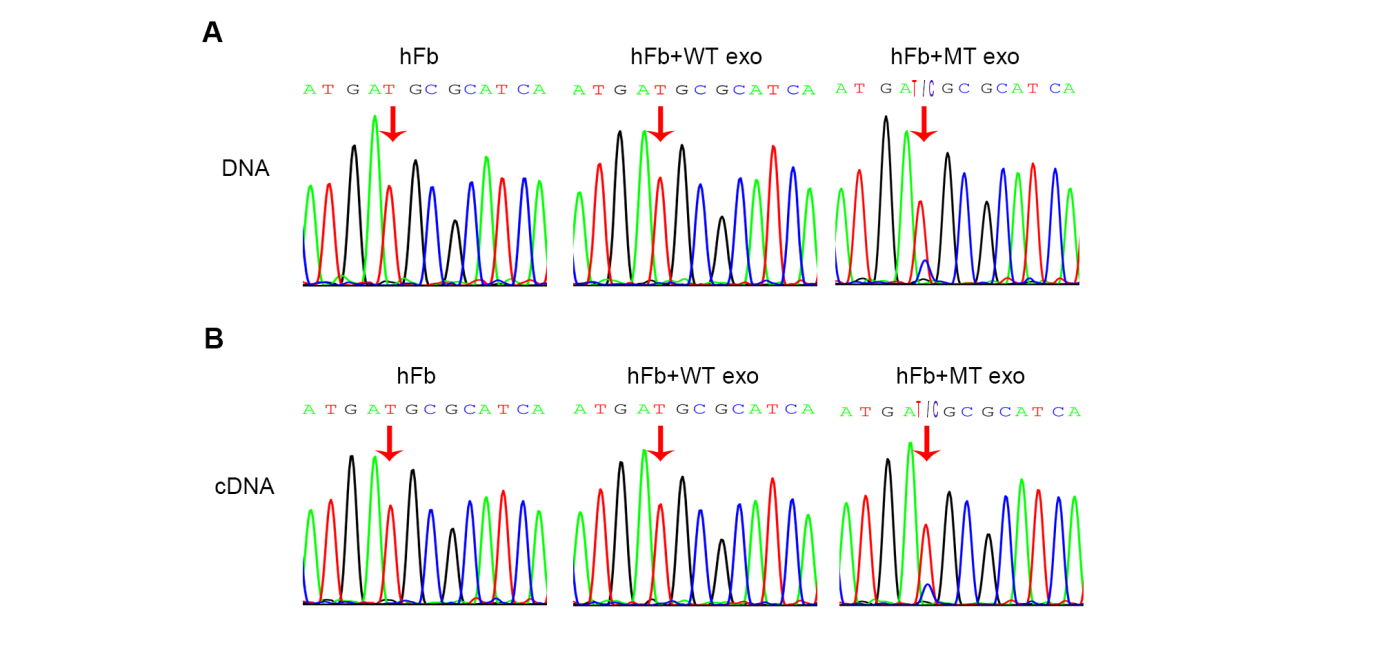


**Figure S4. Horizontal transmission of *PIK3CA*^H1047R^ mutation in MT cell-derived exosomes to hfbs.** A) Sanger sequencing analysis *PIK3CA*^H1047R^ mutation in hFbs DNA co-incubated with WT and MT cell-derived exosomes for 24 h. B) **S**anger sequencing analysis *PIK3CA*^H1047R^ mutation in hFbs cDNA co-incubated with WT and MT cell-derived exosomes for 72 h.


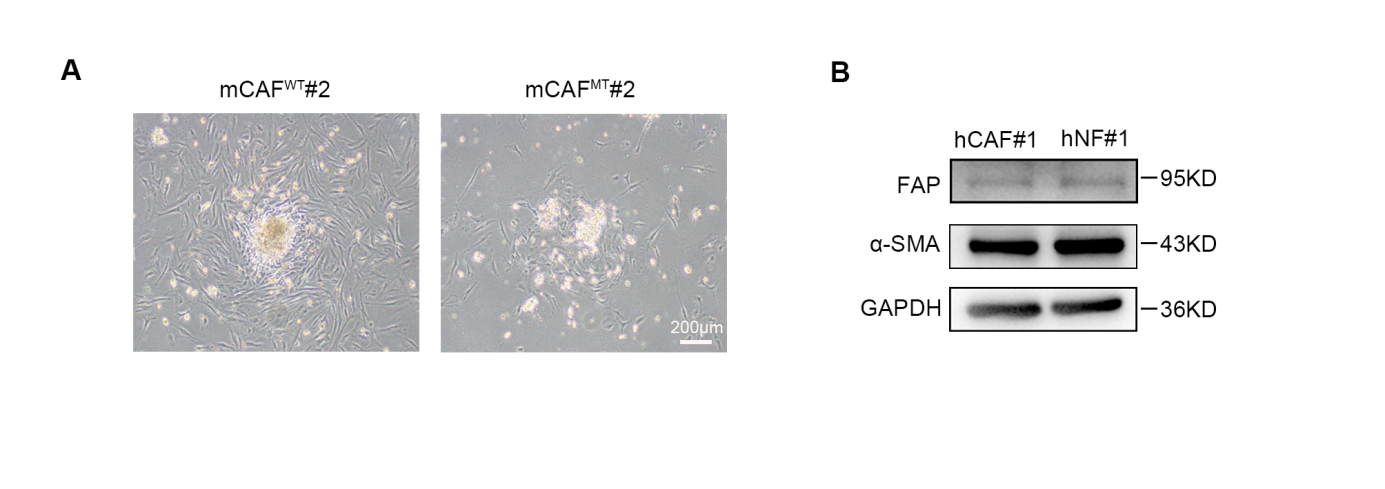


**Figure S5. Identification of fibroblasts after primary isolation.** A) The morphology of primary isolated mCAFs. Scale bar, 200 μm. B) Western blot analysis of FAP and α-SMA expression in the fifth-passage hCAFs and hNFs from one set of paird patient with CRC.


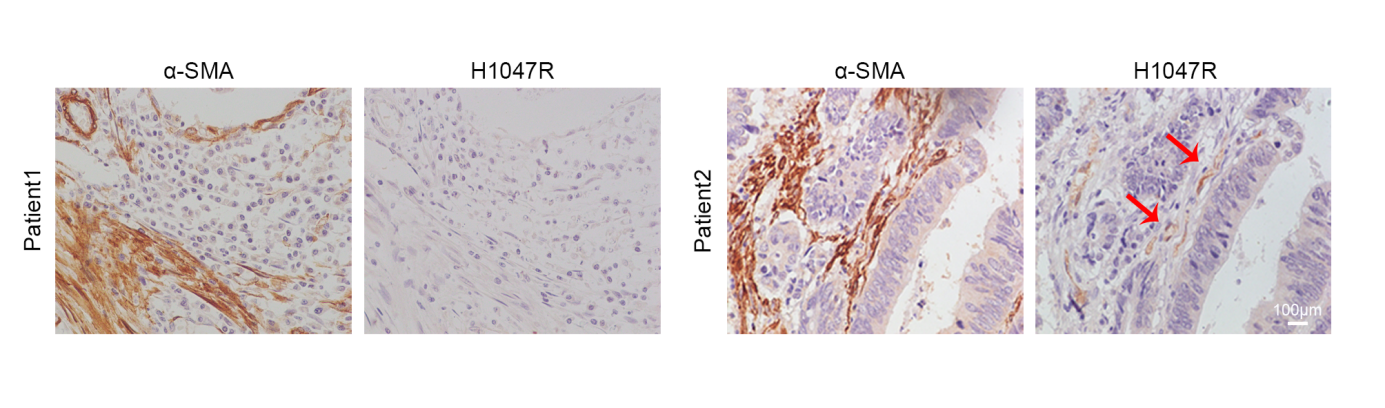


**Figure S6. The expression of H1047R in patients with CRC.** Immunohistochemical analysis of H1047R expression in hCAFs from patient 1 and 2, α-SMA in serial sections served as the hCAFs marker. Scale bar, 100 μm.


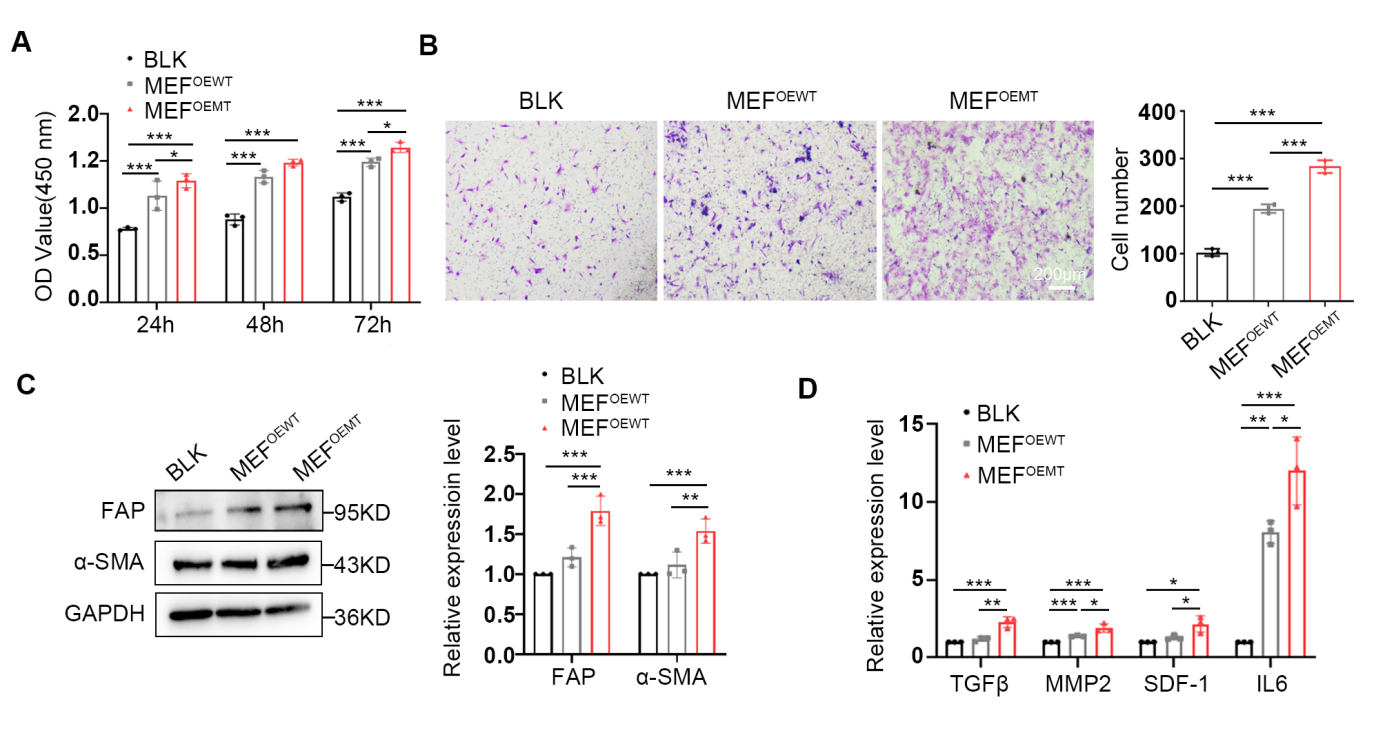


**Figure S7. Overexpressing H1047R activates MEFs into CAFs.** A) CCK8 assay to determine the proliferation of WT and MT-overexpressing MEFs or control cells. Error bars, mean±SD. Two-way ANOVA with multiple comparisons. * *P* < 0.01, *** *P* < 0.0001, *n*=3. B) Transwell assay to determine the migration viability of WT and MT-overexpressing MEFs or control cells. Quantitative analysis is shown in the right panel. Error bars, mean±SD. One-way ANOVA with multiple comparisons. *** *P* < 0.0001, *n*=3. Scale bar, 200 μm. C) Western blot analysis of FAP and α-SMA expression in WT and MT-overexpressing MEFs or control cells. Error bars, mean±SD. Two-way ANOVA with multiple comparisons. ** *P* < 0.001, *** *P* < 0.0001, *n*=3. D) Real-time PCR analysis of TGFβ, MMP2, SDF-1, and IL6 expression in WT and MT-overexpressing MEFs. Error bars, mean±SD. Two-way ANOVA with multiple comparisons. * *P* < 0.01, ** *P* < 0.001, *** *P* < 0.0001, *n*=3.


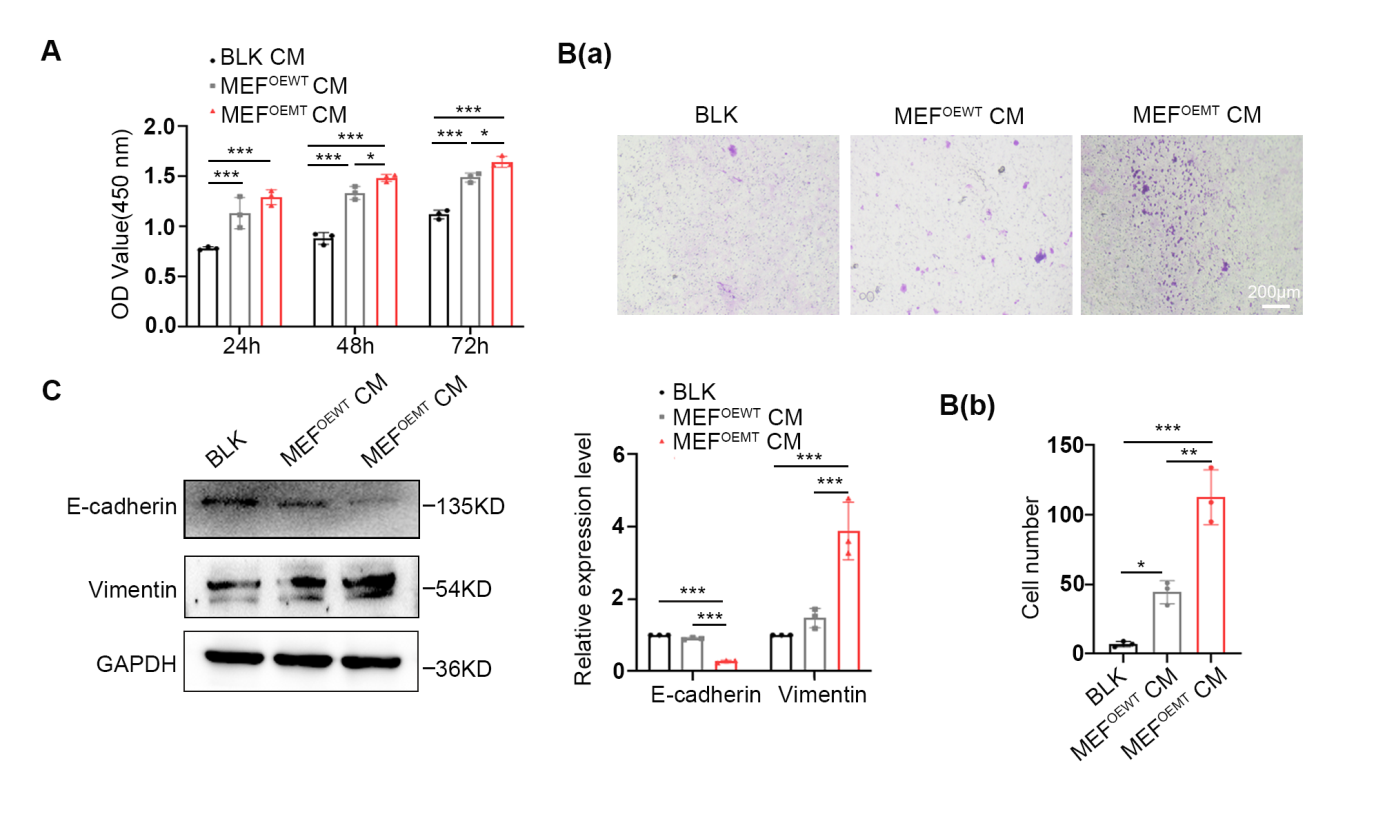


**Figure S8. *PIK3CA*^H1047R^ mutation is associated with CRC metastasis.** A) CCK8 assay to determine WT cells proliferation viability treated with CM from WT and MT-overexpressing MEFs or control cells. Error bars, mean±SD. Two-way ANOVA with multiple comparisons. * *P* < 0.01, *** *P* < 0.0001, *n*=3. B) a). Transwell assay to determine the migration viability of WT cells treated with CM from WT and MT-overexpressing MEFs or control cells. B) b). Quantitative analysis of B(a). Error bars, mean±SD. One-way ANOVA with multiple comparisons. * *P* < 0.01, ** *P* < 0.001, ***p < 0.0001, *n*=3. Scale bar, 200 μm. C) Western blot analysis of EMT markers E-cadherin and Vimentin expression in WT cells treated with CM from WT and MT-overexpressing MEFs or control cells. Quantitative analysis is shown in the right panel. Error bars, mean±SD. Two-way ANOVA with multiple comparisons. *** *P* < 0.0001, *n*=3.


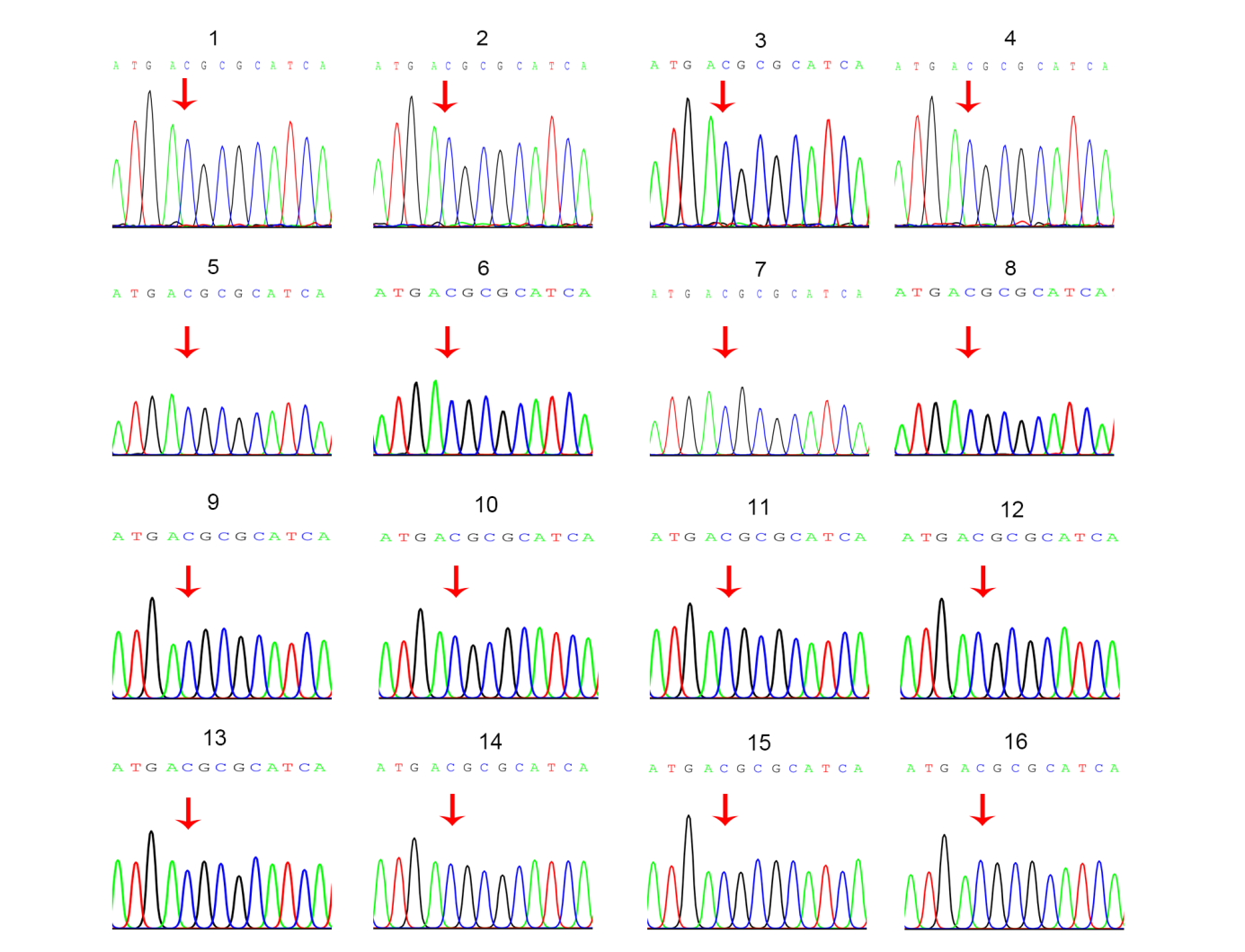


**Figure S9. Serum-derived exosomes from patients with CRC contain *PIK3CA*^H1047R^ mutation.** T-A clonal sequencing analysis of *PIK3CA*^H1047R^ mutation in the serum-derived exosomes from 16 patients with CRC with *PIK3CA*^H1047R^ mutation.


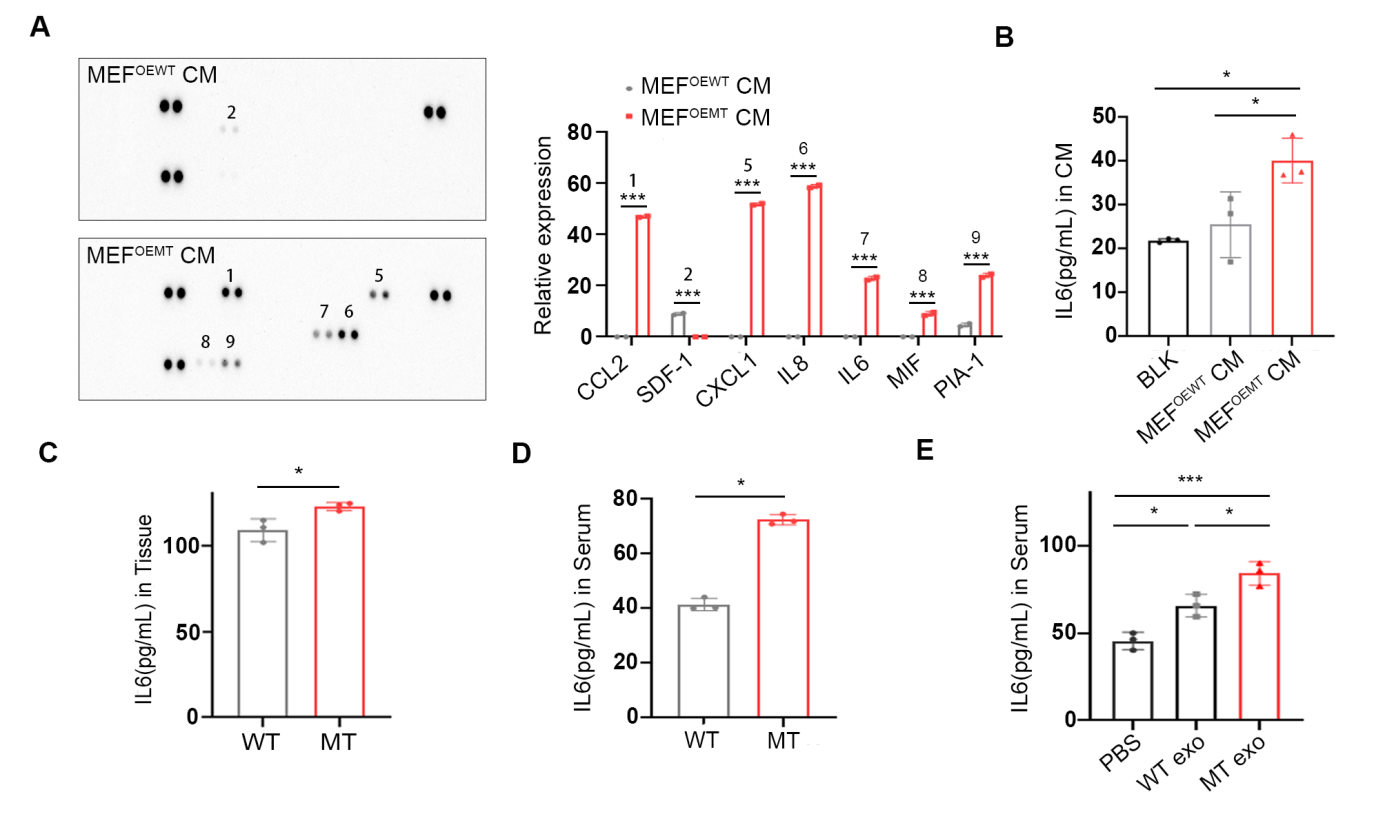


**Figure S10. *PIK3CA*^H1047R^ mutation induces CAFs to secrete elevated levels of IL6.** A) Cytokine Analysis Array System analysis of the CM from WT and MT-overexpressing MEFs. Quantitative analysis is shown in the right panel. Error bars, mean±SD. Two-way ANOVA with multiple comparisons. *** *P* < 0.0001, *n*=2. B) ELISA analysis of IL6 secretion in the CM from WT and MT-overexpressing MEFs or control cells. Error bars, mean±SD. One-way ANOVA with multiple comparisons. * *P* < 0.01, *n*=3. C) ELISA analysis of IL6 expression in tumors from mice with WT and MT cells subcutaneously injection. Error bars, mean±SD. Two-sided Student’s t test. * *P* < 0.01, *n*=3. D) ELISA analysis of IL6 expression in serum from mice with WT and MT cells subcutaneously injection. Two-sided Student’s t test. * *P* < 0.01, *n*=3. E) ELISA analysis of IL6 expression in serum from mice of WT and MT cell-derived exosomes tail vein injection. Error bars, mean±SD. One-way ANOVA with multiple comparisons. * *P* < 0.01, *** *P* < 0.0001, *n*=3.


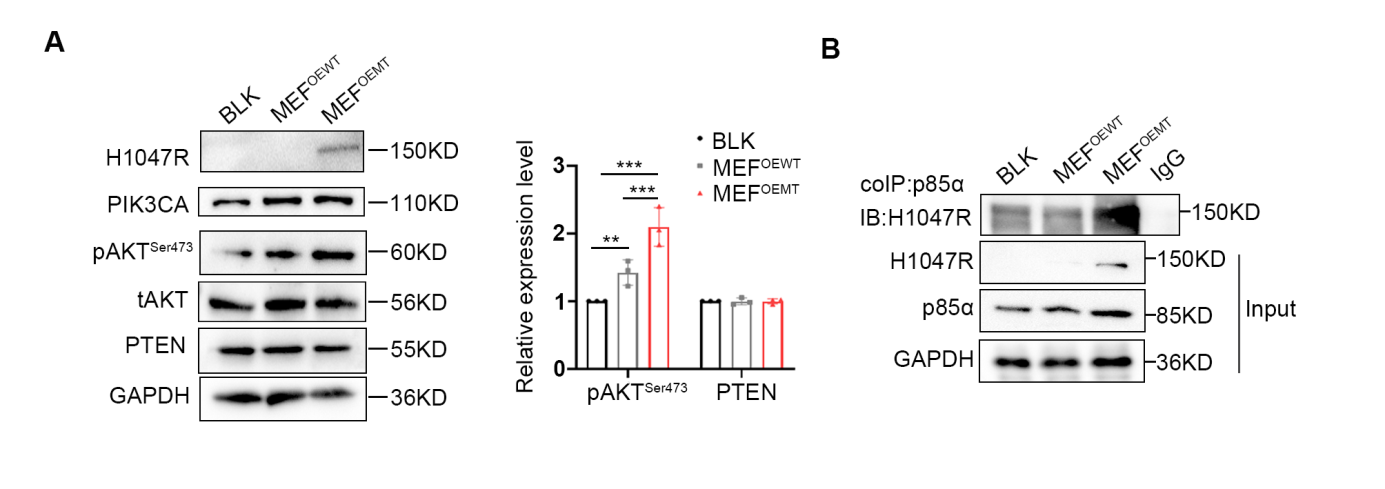


**Figure S11. CAFs promote CRC metastasis through the PI3K/NF-κB/IL6/STAT3 pathway *in vitro*.** A) Western blot analysis of PI3K signal expression in WT and MT-overexpressing MEFs or control cells. Error bars, mean±SD. Two-way ANOVA with multiple comparisons. ** *P* < 0.001, *** *P* < 0.0001, *n*=3. B) Co-IP analyses were performed to examine the exogenous PIK3CA^H1047R^ competitive binding to p85α intracellularly in WT and MT-overexpressing MEFs.


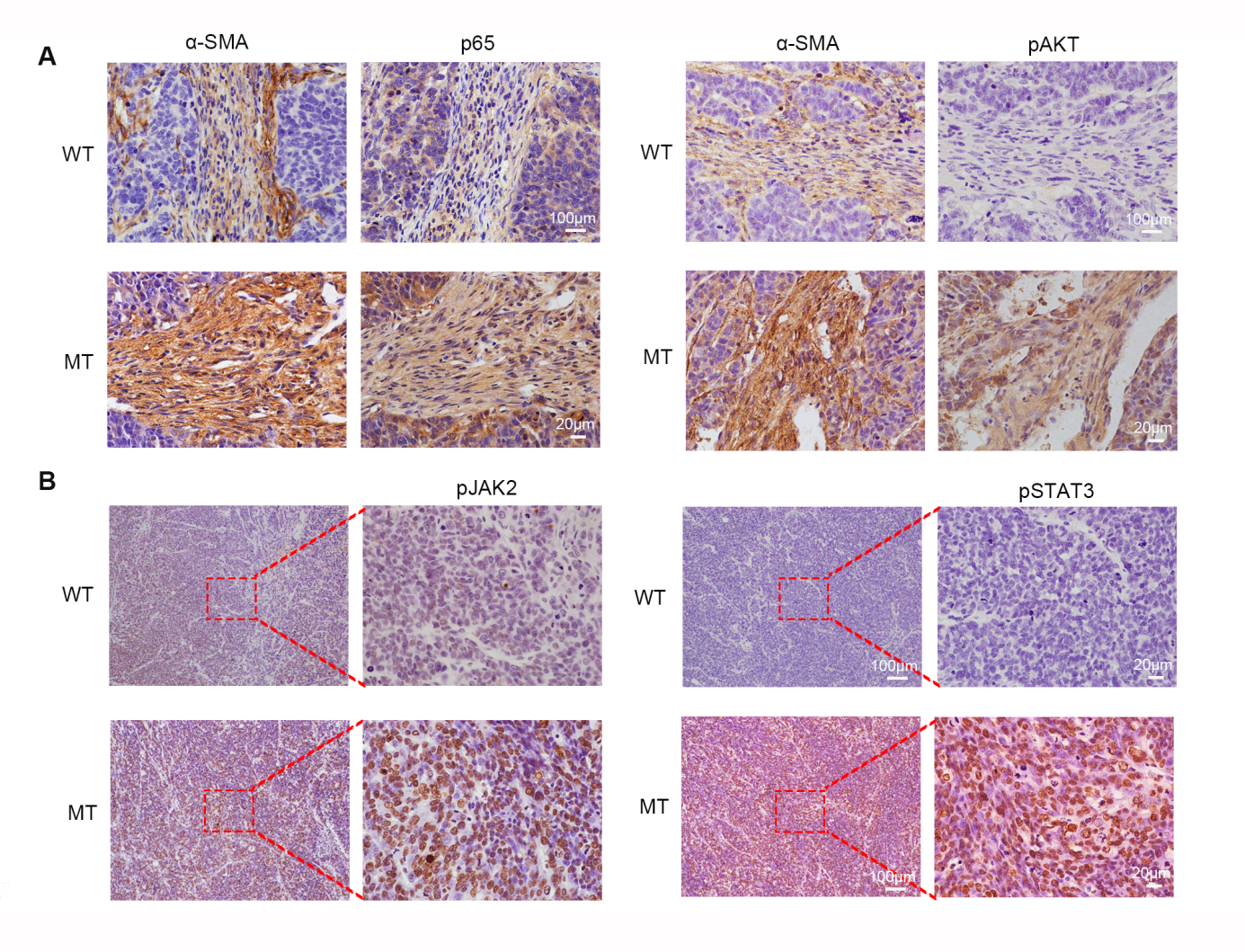


**Figure S12. CAFs promote CRC metastasis through the PI3K/NF-κB/IL6/STAT3 pathway in vivo.** A) Immunohistochemical staining the expression levels of pAKT and p65 in fibroblasts in tissues from mice-bearing WT and MT cells. B) Immunohistochemical staining the expression levels of pJAK2 and pSTAT3 in tumor cells in tissues from mice-bearing WT and MT cells.

Table S2. The antibodies used in this research

| **Western blot** | | |  |  |
| --- | --- | --- | --- | --- |
| **Primary antibody** |  |  |  |  |
| **Protein name** | **Dilution** | **Company** |  |  |
| H1047R | 1:1000 | Arigo#ARG66505 |  |  |
| CD63 | 1:1000 | Proteintech#67605-1-Ig |  |  |
| CD9 | 1:1000 | Proteintech#20597-1-AP |  |  |
| CD81 | 1:1000 | Proteintech#66866-1-Ig |  |  |
| α-SMA | 1:2000 | Proteintech#14395-1-AP |  |  |
| FAP | 1:1000 | Abcam#ab314456 |  |  |
| PTEN | 1:1000 | Proteintech#22034-1-AP |  |  |
| p-AKT | 1:1000 | CST#4060 |  |  |
| AKT | 1:1000 | Immunoway#YT0185 |  |  |
| p-STAT3 | 1:1000 | ZEN#381552 |  |  |
| STAT3 | 1:1000 | ZEN#251611 |  |  |
| p-JAK2 | 1:1000 | ZEN#381556 |  |  |
| JAK2 | 1:1000 | ZEN#R24775 |  |  |
| E-cadherin | 1:1000 | Abcam#ab238099 |  |  |
| Vimentin | 1:2000 | Proteintech#10366-1-AP |  |  |
| GAPDH | 1:10000 | Proteintech#60004-1-Ig |  |  |
| IL6 | 1:1000 | Immunoway#YT5348 |  |  |
| **Secondary antibody** |  |  |  |  |
| Goat anti-mouse IgG HRP | 1:10000 | Proteintech#SA00001-7L |  |  |
| Goat anti-rabbit IgG HRP | 1:10000 | Proteintech#SA00001-4 |  |  |
| **Immunohistochemistry** | | |  |  |
| α-SMA | 1:200 | Proteintech#14395-1-AP |  |  |
| H1047R | 1:200 | Immunoway#YM6978 |  |  |
| IL6 | 1:200 | Immunoway#YT5348 |  |  |
| FAP | 1:200 | Abcam#ab314456 |  |  |
| E-cadherin | 1:200 | Abcam#ab238099 |  |  |
| Vimentin | 1:200 | Proteintech#10366-1-AP |  |  |
| **Immunofluorescence** | | |  |  |
| **Primary antibody** | | |  |  |
| α-SMA | 1:1000 | Proteintech#14395-1-AP |  |  |
| H1047R | 1:200 | Immunoway#YM6978 |  |  |
| IL6 | 1:200 | Immunoway#YT5348 |  |  |
| **Secondary antibody** |  |  |  |  |
| Alexa FluorTM 488 goat anti-rabbit IgG (H+L) | 1:200 | Invitrogen#A-11008 |  |  |
| Alexa FluorTM 555 goat anti-mouse IgG (H+L) | 1:200 | Invitrogen#A-31570 |  |  |
